# Barriers and facilitators to implementing practices for prevention of childhood obesity in primary care: A mixed methods systematic review

**DOI:** 10.1111/obr.13417

**Published:** 2022-01-22

**Authors:** Devashish Ray, Falko Sniehotta, Elaine McColl, Louisa Ells

**Affiliations:** ^1^ Population Health Sciences Institute Newcastle University Newcastle upon Tyne UK; ^2^ School of Clinical and Applied Sciences Leeds Beckett University Leeds UK

**Keywords:** children, guidelines, obesity prevention, primary care providers

## Abstract

Primary care providers (PCPs) have an important role in prevention of excess weight gain in pre‐school children. Guidelines exist to support PCPs' practices. This systematic review of PCPs' practice behaviors and their perceptions of barriers to and facilitators of implementation of guidelines was the first step toward the development of an intervention aimed at supporting PCPs. Five databases were searched to identify qualitative, quantitative, and mixed methods studies which examined PCPs' practice patterns and factors influencing implementation of recommended practices. The convergent integrated approach of the Joanna Briggs Institute (JBI) methodology for mixed methods reviews was used for data synthesis. Following analyses, the resultant factors were mapped onto the Capability, Opportunity, and Motivation model of Behaviour (COM‐B). Fifty studies met the eligibility criteria. PCPs inconsistently implement recommended practices. Barriers and facilitators were identified at the provider (e.g., lack of knowledge), parent (e.g., lack motivation), and organization level (e.g., inadequate training). Factors were mapped to all three components of the COM‐B model: psychological capability (e.g., lack of skills), reflective motivation (e.g., beliefs about guidelines), automatic motivation (e.g., discomfort), physical opportunity (e.g., time constraints), and social opportunity (e.g., stigma). These findings reflect the complexity of implementation of childhood obesity prevention practices.

## 1 INTRODUCTION

Childhood obesity has reached epidemic levels in both developed and developing countries.^1^ In England, around one in four children aged 4–5 years have overweight or obesity, with the prevalence more than double in the most deprived areas compared to the least deprived.^2^ This trend is of concern because childhood obesity is associated with significant adverse effects on physical and psychosocial health in childhood and tends to persist into adulthood, with increased risk of diabetes, heart disease and certain cancers during adult life.^3^ Targeting modifiable risk factors for excessive weight gain during early life with prevention interventions may help in addressing childhood obesity and influencing the inequalities in prevalence.^4^

Primary care provides opportunities for practitioners and parents/caregivers to discuss healthy growth, nutrition, and strategies for prevention of childhood obesity. Several governments and organizations have published guidelines for prevention of childhood obesity in primary care. In England, the National Institute for Health and Care Excellence (NICE)^5^, ^6^, ^7^ and Public Health England (PHE)^8^ have developed guidelines for PCPs who have a role in prevention of childhood obesity. However, it is widely acknowledged that practitioners do not routinely implement guideline recommended practices.^9^ Implementation of guidelines is influenced by a range of factors which may be related to the guideline, the healthcare setting, and the social, cultural, economic, and political context in which PCPs work. These factors are collectively referred to as barriers to and facilitators of implementation, or more broadly as “determinants of clinical behaviours.” Identification, appraisal, and synthesis of the existing evidence regarding PCPs' current practices and their perceptions of factors that influence their practice behaviors can inform the development of strategies and interventions to support PCPs' role, service development, and future research into obesity prevention.

A PCP's behavior can be explained and predicted using the same processes and behavioral models that can be applied to human behavior in general. The Capability, Opportunity, and Motivation model of Behaviour (COM‐B) model of behavior^10^ proposes that interactions between capability, opportunity, and motivation result in the performance of the behavior that in turn influences those three components. Capability is defined as the individual's psychological (e.g., knowledge and communication skills) and physical capability (e.g., physical skills) to engage in the specified behavior. Opportunity refers to factors in the external environment that prompt or enable the performance of the behavior and includes both physical (e.g., resources) and social (e.g., social norms) opportunity. Motivation refers to the brain processes that facilitate the behavior (as a priority over other competing behaviors); they can be reflective (e.g., analytical decision making) or automatic (e.g., habits, and emotional responses, cued by environment). The COM‐B model was used in this review to develop a theoretical understanding of the factors that influence practitioners' practice behaviors. This SR aimed to synthesize the evidence on (1) PCPs' current practices to prevent development of obesity in 0–5 year old children; (2) barriers to, and facilitators of guideline recommended practices as perceived by PCPs; and (3) to map these onto the COM‐B model. This research was published as an abstract in a special supplement of Obesity Reviews in 2020.^11^

## 2 METHODS

Qualitative and quantitative evidence was included in the review to account for the inherent complexity of implementing clinical practices in primary care. The convergent integrated approach, according to the JBI methodology for mixed‐methods reviews, was used for evidence synthesis.^12^ This involved simultaneously integrating and synthesizing quantitative and qualitative data through data transformation. The protocol of this review was registered with the International Prospective Register of Systematic Reviews (CRD42017084067). The review is reported in accordance with the updated Preferred Reporting Items for Systematic Reviews and Meta‐analysis (PRISMA) 2020 reporting guidelines^13^ (presented in Table S1).

### 2.1 Eligibility criteria

For this review, the concept of primary care was based on the World Health Organization (WHO)'s definition of “integrated” primary healthcare: a comprehensive health system which integrates key public health functions (health promotion and preventive care) into existing primary care services.^14^ PCPs were defined as practitioners who work in primary care and provide services including health promotion, disease prevention, patient education and counseling. They included doctors (e.g., general practitioners and general pediatricians) and nurses (e.g., practice nurses, health visitors, pediatric nurse practitioners, maternal and child health nurses, and breastfeeding specialist nurses), community midwives, and community dieticians. A barrier was defined as a factor that hindered implementation of guidelines; a facilitator was defined as a factor that promoted implementation. Eligible studies were primary research studies reporting on (i) implementation/non‐implementation by PCPs of practices recommended for prevention of excess weight in children aged 0–5 year; (ii) behavioral determinants (e.g., PCPs' knowledge, attitudes, and beliefs); and (iii) barriers and facilitators of implementation of practices as perceived by PCPs. Pre‐2002 studies were excluded as UK guidelines for prevention of childhood obesity in primary care were first introduced around this time.^15^ Only published peer‐reviewed papers in English were included. Table 1 summarizes the inclusion and exclusion criteria.

**TABLE 1:** List of inclusion and exclusion criteria

Inclusion criteria	Exclusion criteria
Sample (Population): Primary care practitioners (e.g., doctors, nurses including community nurses, specialty public health nurses, and community midwives) Phenomenon of interest (Intervention): Care provided to 0–5 year olds in primary care settings for prevention of excess weight gain; studies that reported on care involving a broader age group (e.g., 0–18 or 2–18) were included if the age range included 0–5; studies reporting on care provided for breastfeeding mothers; studies that looked into both prevention and treatment were included if data relevant to preventive care could be separated Outcomes: Research reporting on implementation/non‐implementation of recommended practices Research exploring behavioral determinants (e.g., perceptions, attitudes, knowledge, and self‐efficacy) Research reporting on barriers to and/or facilitators of implementation of practice Research design: Quantitative (survey studies); Qualitative, Mixed methods Search limits: English language studies from January 2002 onward	Sample (Population): Non‐healthcare professionals, parents, students, social workers, managers, project directors Phenomenon of interest (Intervention) Research focuses exclusively on treatment rather than prevention of childhood obesity Studies set exclusively outside primary care (e.g., hospitals) Preventive care exclusively for children >5 years of age Outcomes Studies that reported on outcomes of an implementation intervention or quality improvement project Research design: Studies that were not primary research (e.g., review, commentary, or opinion paper) Search limits: Time period: papers published prior to January 2002 Not published in English

### 2.2 Search strategy

A three‐step strategy, as recommended by the JBI, was used to identify eligible papers.^16^ Sets of search terms using combinations of key words were used across the following categories: primary care, prevention of childhood obesity, practice behaviors, phenomenon of interest (practice patterns, barriers and facilitators, knowledge, attitudes, beliefs) and research designs. Five databases (Medline, Embase, British Nursing Index, CINAHL, and PsycINFO) were searched by DR (lead reviewer) from 2002 to March 2018 and updated on 21 April 2021 to identify eligible papers. The search strategy was initially developed in MEDLINE with support from a specialist librarian, and appropriately tailored for use for the other databases, and piloted before final searches were run. The final MEDLINE search strategy is presented in Figure S1.

### 2.3 Study screening and selection process

**FIGURE 1:**
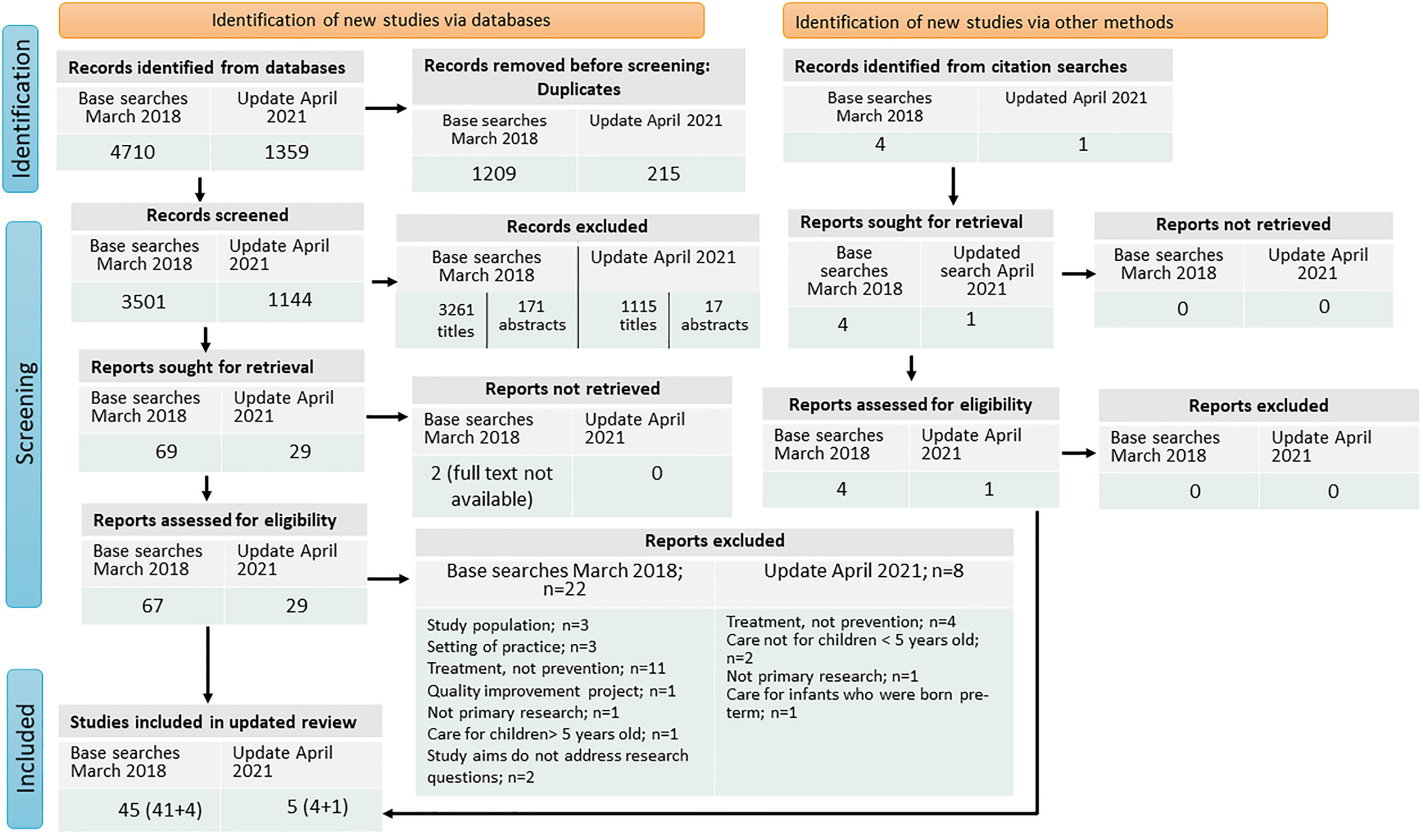
Preferred Reporting Items for Systematic Reviews and Meta‐analysis (PRISMA) 2020 flow diagram, tailored for this systematic review

Eligibility screening of titles and abstracts was undertaken by DR. An overview of the study screening and selection process of the original search and the updated search are presented using a tailored PRISMA 2020 flow diagram^17^ (see Figure 1). The reasons for exclusion of full text papers were documented by DR and independently verified by a researcher experienced in conducting SRs. The most common reason for excluding full text papers was that the study focused exclusively on treatment of children having obesity.

### 2.4 Quality appraisal

DR assessed the quality of all the papers using JBI critical appraisal checklists^18^, ^19^ that are specific to the research methodology. Co‐authors acted as the second reviewers. DR's quality assessment work on 50% of the qualitative and mixed methods papers was checked by FS for accuracy. EM verified DR's quality assessment of 25% of the quantitative papers. Any inaccuracies/discrepancies were resolved through discussion. The assessment process was not used to exclude papers but as a broad guide to provide a context for interpreting the findings.

### 2.5 Data extraction

Data on aims, study design, participants' characteristics, data collection methods, theoretical framework used (if any), and main findings (i.e., survey results, themes identified by study authors, and participant quotations) were extracted from each paper, using tools available from JBI.^20^, ^21^ Data extraction of all the papers was undertaken by DR, and 20% were checked for accuracy by a second reviewer (LE). Any inaccuracies/discrepancies were resolved through discussion.

### 2.6 Data synthesis

**FIGURE 2:**
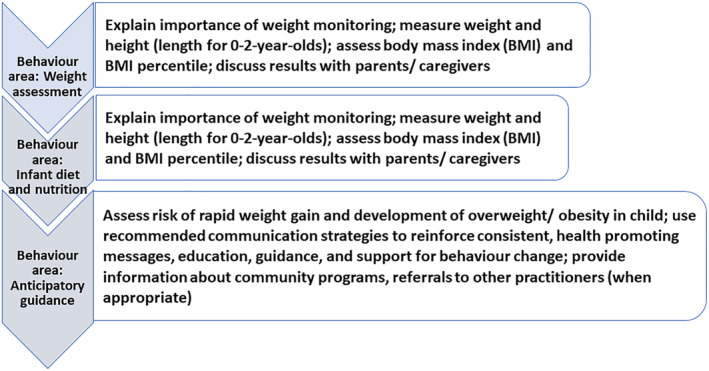
Behavior areas for primary care providers, based on guidelines published by National Institute for Health and Care Excellence and Public Health England

As specified in JBI's convergent integrated approach for mixed methods review,^16^ the quantitative data extracted from survey studies and quantitative component of mixed methods studies were “qualitised” through narrative interpretation of the findings into textual descriptions. Subsequently, the “qualitised data” was assembled with the qualitative data extracted from qualitative studies and the qualitative component of mixed methods studies. To guide the synthesis of the evidence related to PCPs' practice behaviors, three “behaviour areas” were identified based on the NICE^5^, ^6^, ^7^ and PHE^8^ guidelines for prevention of excess weight development in 0–5 year olds. The behavior areas were developed by grouping the guideline recommendations into themes and identifying the clinical behaviors that are expected to be carried out by PCPs within each area during their interactions with children and parents (Figure 2). Thematic synthesis of the assembled data was carried out by DR using an iteratively developed coding frame. The themes and categories were refined through discussion with review team members (FS, LE, and EM) at multiple meetings to ensure that they accurately reflected the data. A narrative account of the synthesis was prepared, and quotations were taken from the studies to illustrate the findings. Subsequently, the findings were mapped onto the COM‐B model by DR following expert guidance^22^ and in consultation with the review team.

## 3 RESULTS

### 3.1 Summary of the studies

Fifty studies: 21 qualitative, 24 quantitative (cross‐sectional surveys), and 5 mixed methods studies met the eligibility criteria. Of these, 45 studies were identified in the original search, with five additional studies identified in the updated search. The studies were conducted in the USA (*n* = 25), continental Europe (*n* = 9), the United Kingdom (*n* = 8), Australia (*n* = 5), Canada (*n* = 2), and New Zealand (*n* = 1). The settings most frequently described in the USA‐based studies were family medicine and well‐child clinics that were either physician‐owned or affiliated to large provider organizations, or to academic/hospital‐affiliated primary care services. The settings described in the remaining studies included child health centers affiliated to community health services (provided by local government), home visiting, and general practices. Only two studies^23^, ^24^ reported on experiences of PCPs working in rural settings. Participants were exclusively from the nursing profession (e.g., family nurse practitioners, child health nurses, health visitors, pediatric nurse practitioners) in 16 studies; in 13 studies, they were exclusively doctors (e.g., general practitioners, family physicians, pediatricians); and in the remaining 21 studies, the samples were mixed (e.g., doctors and nurses with different levels of training and speciality roles). A small number of studies using mixed samples, identified community midwives (3 studies), community dietitians/nutritionists (3 studies), breastfeeding counselors (2 studies), lactation consultants (3 studies), and social worker (1 study) among study participants. Thirty‐one studies reported on PCP's current practices. Barriers and/or facilitators were reported by 43 studies; of these, 25 studies also reported on PCPs' practices. Data from the studies were tabulated to allow comparison of the country of origin, key objectives, participant characteristics, service user group, study design, and the primary care setting. This information is presented in Table S2.

Only eight studies used a psychological theory or model to guide the research. The majority of the qualitative studies reported using purposive sampling, independent coding by multiple researchers and using consensus meetings to resolve discrepancies. However, only six studies reported on the influence of the researcher(s) on the research. The majority of survey studies used restricted sampling frames (very few studies used national databases) and convenience (not random) sampling to recruit participants, thus limiting the potential for generalizability of the findings. Most studies provided information on response rates, used appropriate analytic methods, and acknowledged the potential for self‐ selection bias and self‐reporting bias as methodological limitations. A cross‐study summary of the quality appraisal is presented in Table 2 (qualitative studies) and Table 3 (survey studies). The full quality appraisal of the individual studies is available upon request.

**TABLE 2:** Quality appraisal of qualitative studies (*n* = 21) and qualitative component of mixed methods studies (*n* = 5)

Quality appraisal checklist item	Percentage meeting criteria across studies (*n* = 26)
1. Philosophy congruent	16
2. Objective congruent	100
3. Data collection congruent	100
4. Data analyses congruent	96
5. Interpretation of results	96
6. Theory or cultural stance	32
7. Researcher reflexivity	24
8. Participant representation (direct quotations)	96
9. Ethical consideration	92
10. Conclusions of the research	100

**TABLE 3:** Quality appraisal of quantitative studies (*n* = 24) and quantitative component of mixed method studies (*n* = 5)

Quality appraisal checklist item	Percentage meeting criteria across studies (*n* = 29)
1. Appropriate sampling frame	90
2. Appropriate sampling strategy	96
3. Sample size calculation	21
4. Setting and participants information	100
5. Were valid methods used?	38 (unclear for 41% of the studies)
6. Were outcomes measured reliably?	38 (unclear for 41% of the studies)
7. Was questionnaire piloted?	36
8. Response rate information	90
9. Were potential biases discussed?	90
10. Were appropriate analysis methods used?	96 (unclear for 1 study)

Three broad organizing themes emerged from the synthesis: PCPs' practice implementation behaviors, and barriers to‐, and facilitators of implementation. The barriers and facilitators were categorized at the PCP, family, and organizational level. No new themes or new barriers/facilitators were identified in by adding the five additional papers (published between 2019 and 2021) to update the review. The findings are discussed in the following sections with participant identifiers. An overview of the barriers and facilitators with indicative quotes is presented in Tables S3 (barriers) and S4 (facilitators).

### 3.2 Primary care provider's practice implementation behaviors

#### 3.2.1 Weight and growth assessment

Twenty‐one studies reported on weight assessment practices. PCPs generally relied on height and weight growth charts,^25^, ^26^, ^27^, ^28^, ^29^, ^30^, ^31^, ^32^, ^33^, ^34^ or simple visual inspection^25^, ^26^, ^32^, ^33^, ^35^, ^36^ to assess a child's weight status and monitor weight gain over time. There were geographic differences in the use of reference charts used by PCPs. The reference most commonly cited in the USA‐based studies were the CDC (Centre for Disease Control) growth chart^37^ (for children over age 2) and the WHO standards^38^ (for children under age 2) whereas studies from other countries reported the use of national standards based on WHO standards. Although the BMI chart was regarded as a facilitator of conversations about weight,^25^, ^26^, ^39^, ^40^, ^41^ the routine use of BMI for 2–5 year‐olds (and weight‐for‐length charts for children under 2) was low, with roughly a third of PCPs never using BMI (and weight‐for‐length for <2),^25^, ^29^, ^32^, ^33^, ^36^, ^42^, ^43^, ^44^, ^45^, ^46^, ^47^, ^48^ or using it selectively, for example, only if PCPs were concerned.^26^, ^36^, ^47^ One USA‐based study found that the routine use of BMI by pediatricians at well‐child visits has increased over the past decade.^49^ However, many PCPs who reported they regularly measured BMI were not aware of the guidelines for classifications applied for overweight/obesity.^28^, ^31^, ^33^, ^42^, ^50^

Low use of BMI was associated with PCPs' lack of familiarity with BMI,^25^, ^26^, ^28^, ^31^, ^32^, ^33^, ^34^, ^35^, ^42^, ^48^, ^50^, ^51^, ^52^ a lack of agreement with the validity and predictive potential of BMI in very young children,^25^, ^26^, ^35^, ^48^, ^51^, ^53^ lack of access to automatic BMI calculators,^26^, ^47^ parents' lack of familiarity with BMI charts,^32^, ^36^ and lack of time.^32^, ^33^, ^36^, ^47^, ^53^, ^54^ BMI use was reportedly high in settings where PCPs had access to tools and electronic medical record systems (which enabled automatic calculation and plotting of BMI percentile values) and support staff for screening.^28^, ^32^, ^33^, ^49^ Role‐specific specialist training, obesity training, familiarity with BMI guidelines and the belief that prevention efforts will produce positive outcomes were identified as facilitators of BMI use.^32^, ^33^, ^48^, ^49^, ^53^

#### 3.2.2 Breastfeeding support

The data on PCPs' breastfeeding support practices was limited. Although most PCPs believed that supporting breastfeeding was an important part of their role,^25^, ^55^, ^56^, ^57^, ^58^ many PCPs did not routinely discuss and provide breastfeeding advice during antenatal and postnatal visits, or assist mothers with specific breastfeeding problems.^57^, ^58^ Only a minority reported having observed a new mother breastfeeding (a guideline recommendation) and many had never counseled mothers about infant feeding methods, assisted mothers with breastfeeding techniques, or managed lactation problems.^56^, ^58^

All PCP groups reported that they felt unprepared to support the needs of breastfeeding mothers.^56^, ^58^, ^59^, ^60^, ^61^ PCPs attributed their lack of knowledge and skills for managing breastfeeding problems to lack of education and training on breastfeeding management. Importantly, many GPs and pediatricians admitted they lacked competence in key topics (e.g., prescribing to breastfeeding mothers; inadequate weight gain in breastfed infants) where other practitioners (e.g., nurses and midwives) may regard them as an expert for specialist referral.^30^, ^61^ Many PCPs acknowledged that they relied on information they had gained anecdotally from colleagues or from their personal or their spouses' breastfeeding experiences.^56^, ^58^, ^61^, ^62^ PCPs expressed concern that this could lead to some PCPs offering advice that ran counter to recommendations and result in mothers receiving conflicting and incorrect messages.^55^, ^59^, ^60^, ^61^, ^62^

PCPs stressed the importance of supporting women with their “choice” and not being perceived by mothers and their own peers as being coercive.^61^, ^62^ Some PCPs considered breastfeeding as difficult and “exhausting” and believed that bottle feeding was perceived as an easier option by some mothers.^34^, ^55^, ^63^ PCPs believed their influence in promoting breastfeeding is limited because mothers experience various barriers to breastfeed^34^, ^55^, ^62^, ^63^; these were described as cultural norms around breastfeeding, mothers' lack of knowledge and confidence in breastfeeding, previous negative breastfeeding experiences, lack of timely support from healthcare services, family members and peers.

#### 3.2.3 Providing anticipatory guidance

There was wide variation in the manner and extent to which PCPs discussed weight related topics with parents. For example, in one study, roughly 80% of PCPs reported that they routinely counseled children/parents on lifestyle behaviors during most or all visits^64^ while another study found that around 75% of PCPs did *not* discuss healthy eating behaviors until after the child's 12‐month visit.^65^ Infant/toddler weight was viewed as a sensitive topic. PCPs found it difficult to raise the topic of weight due to personal discomfort,^24^, ^25^, ^26^, ^27^, ^34^, ^50^, ^51^, ^52^, ^54^, ^66^ fear of offending parents^30^, ^39^, ^40^, ^41^, ^42^, ^47^, ^48^, ^54^, ^64^, ^67^ and previous experience of negative reactions from parents (angry, upset).^25^, ^26^, ^34^, ^40^, ^53^, ^68^

PCPs less frequently discussed healthy eating and physical activity with parents of infants (0–2 year olds) and pre‐school children (2–5 year olds) as compared to school age children.^25^, ^27^, ^30^, ^31^, ^32^, ^43^, ^50^, ^51^, ^65^, ^67^, ^69^ The frequency of counseling also varied depending upon the topic^25^, ^31^, ^44^, ^45^, ^49^, ^65^, ^68^, ^70^; overall, diet and eating behaviors were more frequently discussed than other behaviors that PCPs identified as important risk factors for childhood obesity such as physical activity, television viewing, parenting styles, and parent and child motivation to change. PCPs' counseling about healthy weight mainly involved providing advice about nutrition.^25^, ^26^, ^30^, ^39^, ^42^, ^47^, ^51^, ^64^ However, the focus of dietary advice was generally about the contents of a healthy infant diet and less about infant feeding practices (e.g., responsive feeding).^25^, ^47^ Further, PCPs tended to provide “blanket” nutritional advice and not discuss specific diet and nutrition topics; also, they were more likely to discuss fruit and vegetable consumption than consumption of sugary drinks, fast foods, and energy dense foods.^34^, ^42^, ^44^, ^64^, ^65^, ^66^, ^70^ PCPs lacked awareness of the importance of physical activity in young children^25^, ^31^, ^50^, ^65^, ^67^, ^68^ and placed low priority on raising the topic.^25^, ^34^, ^71^ Children's TV viewing and electronic screen time were also infrequently addressed.^25^, ^31^, ^32^, ^52^, ^64^, ^65^, ^71^, ^72^

### 3.3 Barriers to implementation

#### 3.3.1 Primary care providers related factors

Deficits in knowledge to support breastfeeding women,^55^, ^56^, ^58^, ^59^, ^60^, ^61^ BMI thresholds for classification of overweight and obesity,^28^, ^31^, ^33^, ^35^, ^42^, ^50^, ^54^ risk factors for excess weight gain in infants,^25^, ^30^, ^35^, ^39^ and recommendations for diet, physical activity and screen time for children^24^, ^31^, ^32^, ^33^, ^35^, ^39^, ^50^, ^72^, ^73^ were identified. Perceived lack of skills in raising the topic of weight and related behaviors was frequently reported.^25^, ^30^, ^34^, ^39^, ^40^, ^43^, ^50^, ^51^, ^52^, ^64^, ^68^, ^73^ PCPs felt it was particularly difficult to discuss weight related topics with parents who were regarded as having overweight.^27^, ^31^, ^40^, ^50^, ^73^

PCPs' views about how guidelines fitted with their role and responsibilities appeared to reflect their professional status and level of training. Nurses believed that providing advice about infant feeding was integral to the role of specially trained nurses (e.g., health visitors) who work closely with mothers and infants.^26^, ^30^, ^39^, ^50^, ^73^ However, health visitors did not always see themselves as the experts.^39^, ^67^ Doctors described their role is primarily around identification and management of children who were having overweight.^30^, ^47^, ^74^

PCPs cited the role of obesogenic factors in the environment and expressed skepticism about the effectiveness of their prevention efforts.^23^, ^25^, ^28^, ^32^, ^33^, ^48^, ^50^, ^64^, ^65^, ^66^, ^68^, ^72^, ^73^, ^74^ Many PCPs were reluctant to identify 0–2‐year‐olds as having overweight^25^, ^27^, ^30^, ^39^, ^41^, ^43^, ^48^, ^51^, ^67^, ^69^ and felt it was inappropriate to intervene if the child's weight had just crossed over into the range for overweight.^26^, ^39^, ^51^, ^52^, ^65^ PCPs were less likely to implement a specific guideline if they perceived the recommendation was not based on sound evidence,^25^, ^47^, ^51^, ^52^, ^72^ or restrictive of their professional autonomy.^23^, ^27^, ^74^ Guidelines were viewed as advisory rather than prescriptive and PCPs justified their decision to deviate from the guidelines to adopt a parent‐centered approach.^25^, ^27^, ^30^, ^35^, ^39^, ^52^, ^67^ For example, PCPs considered it inappropriate to delay weaning for all infants until they are 6 months old.^25^, ^30^, ^39^, ^41^, ^48^, ^52^ PCPs were less likely to implement practices that required discussion on topics that they believed could upset the parents,^24^, ^27^, ^28^, ^29^, ^51^, ^52^, ^53^, ^54^, ^66^ and damage the practitioner‐parent relationship.^30^, ^39^, ^40^, ^41^, ^42^, ^47^, ^48^, ^52^, ^64^, ^67^

#### 3.3.2 Parental factors

PCPs described parental practices and beliefs as important risk factors for obesity in pre‐school children; these included unhealthy infant feeding practices^24^, ^25^, ^26^, ^27^, ^30^, ^31^, ^34^, ^39^, ^41^, ^45^, ^51^, ^65^, ^67^ and parental misperceptions of healthy child weight.^25^, ^34^, ^39^, ^40^, ^41^, ^53^, ^67^ PCPs linked these factors to parents' lack of knowledge and poor parenting skills,^34^, ^36^, ^41^, ^42^, ^51^, ^54^ lack of cooking skills,^25^, ^26^, ^27^, ^39^ influence of peers and grandparents,^25^, ^30^, ^34^, ^54^, ^67^ and cultural norms that influence parents' views about healthy infant weight gain.^25^, ^26^, ^34^, ^39^, ^40^, ^41^, ^51^, ^53^, ^67^, ^68^ PCPs believed that parents lack concern and motivation to change^23^, ^25^, ^26^, ^27^, ^31^, ^34^, ^35^, ^39^, ^40^, ^41^, ^42^, ^43^, ^45^, ^50^, ^51^, ^54^, ^64^, ^65^, ^66^, ^67^, ^68^, ^72^, ^73^; parents having overweight and with (assumed) unhealthy lifestyle behaviors were perceived as particularly unconcerned about childhood overweight and not likely to engage with practices recommended for child healthy weight.^27^, ^31^, ^34^, ^39^, ^41^, ^42^, ^50^, ^52^, ^73^ Excess weight gain during early childhood was viewed primarily as a matter of parental responsibility; some PCPs described parents as poor role models and apportioned blame on them.^25^, ^35^, ^51^, ^67^

Socioeconomic and environmental factors were identified as important risk factors and barriers for parents (access to healthy foods, time constraints for working parents).^24^, ^30^, ^34^, ^39^, ^41^, ^42^, ^45^, ^50^, ^51^, ^52^, ^53^, ^54^, ^73^ PCPs in the USA reported that families who do not have insurance that covers obesity preventive care costs (most do not) are unlikely to access care because of concerns about cost.^24^, ^32^, ^42^, ^45^, ^64^

#### 3.3.3 Organization‐level factors

PCPs' practice setting was an important influence in shaping their perceptions about capability. Implementation was hindered when PCPs perceived a lack of support for the PCP's role, lack of strong leadership and poor inter‐disciplinary cooperation.^23^, ^25^, ^27^, ^35^, ^50^, ^52^, ^59^, ^60^, ^63^, ^73^, ^74^ All PCP groups reported there was insufficient time to sensitively discuss weight related topics.^23^, ^24^, ^25^, ^27^, ^31^, ^32^, ^33^, ^34^, ^35^, ^36^, ^39^, ^41^, ^42^, ^50^, ^52^, ^55^, ^63^, ^64^, ^65^, ^66^, ^72^, ^73^, ^74^ Some PCPs expressed concerns about allocation of funding and resources to support the implementation of protocols.^23^, ^63^, ^74^ Other reported barriers included lack of training in breastfeeding support,^56^, ^58^, ^59^, ^60^, ^61^, ^62^ childhood obesity prevention^30^, ^34^, ^35^, ^39^, ^64^ and communication skills^24^, ^25^, ^30^, ^32^, ^35^, ^39^, ^50^, ^64^, ^73^; and lack of practice tools (e.g., clinical decision making)^25^, ^35^, ^39^, ^45^, ^48^, ^50^, ^65^, ^66^, ^73^ and educational materials for parents.^25^, ^34^, ^45^, ^65^, ^73^ PCPs identified various resource needs; these included practice tools to enhance their capability and performance^24^, ^25^, ^32^, ^33^, ^34^, ^36^, ^48^, ^64^, ^68^, ^70^, ^73^ (e.g., BMI charts showing risk stratification and links to intervention strategies). PCPs who worked in rural settings experienced implementation of guidelines as particularly demanding due to few community resources and very limited access to support from specialists or community‐based programs.^23^, ^24^

The lack of continuity of care was perceived as a barrier by some PCPs^24^, ^25^, ^55^, ^62^ because it prevented the development of positive practitioner‐parent relationships and increased the possibility of the parent receiving conflicting advice during contacts. PCPs also cited organizational policies which resulted in gaps in care (e.g., for breastfeeding women during early postpartum period)^55^, ^62^ and limited opportunities for contact with pre‐school children^24^, ^26^, ^66^, ^74^ as important barriers and recommended the provision of additional services to fill existing gaps in care.^24^, ^55^ Some PCPs expressed concern about limited access to specialists (such as dieticians) and community‐based obesity prevention programs.^23^, ^24^, ^25^, ^32^, ^35^, ^39^, ^64^, ^65^, ^73^, ^74^ Lack of collaboration between physicians and nurses^27^, ^30^, ^35^, ^41^, ^67^, ^74^ and lack of support from peers or superiors^23^, ^27^, ^35^, ^50^, ^52^, ^61^, ^73^ was also reported. Nurses spoke of lack of support from doctors in their clinical decisions^26^, ^39^, ^41^, ^52^ and emphasized the importance of feeling confident that the doctors will support their decisions.^26^, ^41^, ^74^

### 3.4 Facilitators of implementation

#### 3.4.1 Primary care provider‐level factors

PCPs' competence and perceived confidence,^23^, ^32^, ^33^, ^44^, ^49^, ^70^ role‐specific specialist education and training (e.g., pediatricians and pediatric nurses),^28^, ^29^, ^35^, ^36^, ^44^, ^45^, ^50^, ^56^, ^59^, ^64^ participation in obesity training^32^, ^33^, ^70^ and breastfeeding training,^56^, ^57^ familiarity with guidelines^32^, ^33^ and greater experience of working with children and mothers^30^, ^34^, ^53^, ^59^, ^60^ were all identified as facilitators of practice. PCPs who believed that their role in prevention of childhood obesity was important reported positive attitudes and intention to implement recommended practices.^23^, ^26^, ^31^, ^33^, ^39^, ^47^, ^54^, ^55^, ^58^, ^62^, ^73^ Motivated PCPs used approaches that facilitated implementation; these included using tactful language to discuss potentially sensitive topics, focusing on overall health and well‐being rather than on weight, framing having overweight as a societal issue, and using the BMI chart to raise the topic of weight, diet and feeding practices.^25^, ^26^, ^31^, ^35^, ^39^, ^40^, ^41^, ^53^, ^54^ Several studies^29^, ^44^, ^56^, ^69^, ^70^, ^72^ reported that female PCPs (regardless of their job role and specialist education) were more knowledgeable of guidelines, reported higher levels of self‐efficacy and positive outcomes of their prevention efforts, and compliance with recommended practices than their male counterparts.

#### 3.4.2 Parent‐level factors

A positive relationship between the PCP and the family^25^, ^39^, ^40^, ^47^, ^66^, ^74^ and parental concern about childhood overweight^26^, ^49^ were identified as facilitators. PCPs believed that when parents themselves raised concerns about their child's weight, they were more likely to engage with PCPs and comply with recommendations.

#### 3.4.3 Organization‐level factors

Implementation was enabled by perception of role support from the organization^23^, ^33^, ^64^, ^73^ and access to training opportunities.^32^, ^33^, ^34^, ^45^, ^53^, ^62^, ^70^, ^72^ PCPs identified availability of sufficient time and support staff,^24^, ^25^, ^32^, ^55^ access to specialist staff (dieticians and breastfeeding support staff) and local community based family‐centered obesity prevention programs^25^, ^45^, ^70^ as potential facilitators of implementation. Some PCPs believed that a uniform coherent approach to obesity prevention^23^, ^26^, ^41^, ^74^ and closer working between physicians and nurses^26^, ^41^, ^53^, ^62^ can help improve the quality of preventive care.

### 3.5 Theoretical analysis of the barriers and facilitators

**FIGURE 3:**
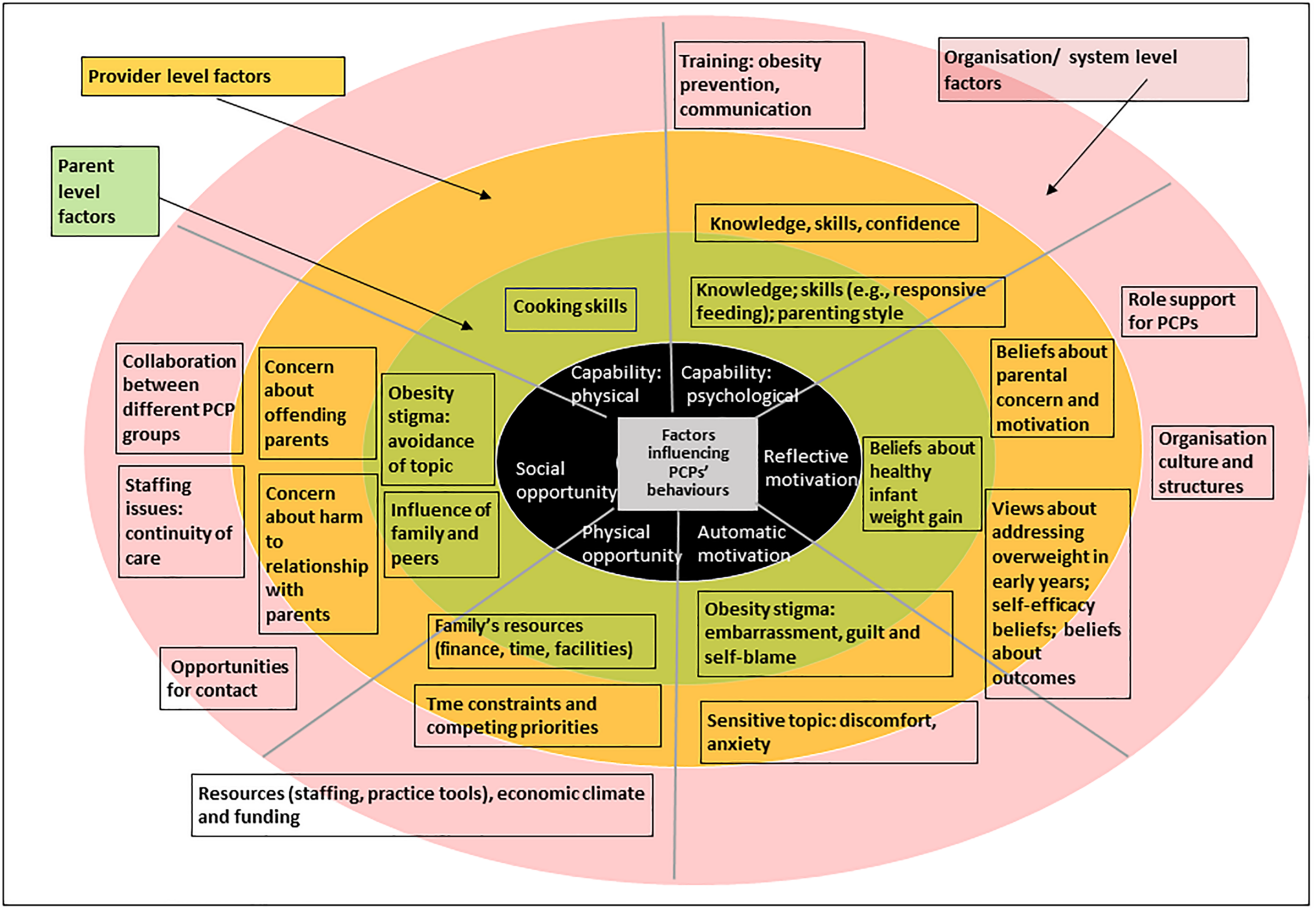
Key factors at organization, provider and patient levels mapped on to the sub‐components of the Capability, Opportunity, and Motivation model of Behaviour (COM‐B) model; factors that were identified as facilitators were the antithesis of the reported barriers

The mapping of the different barriers and facilitators to the COM‐B components (Figure 3) is presented in Tables 4 (barriers) and 5 (facilitators). The mapping revealed that most barriers and facilitators could be allocated to a specific sub‐component of the model; however, some findings could be categorized in more than one sub‐component. For example, PCP's beliefs about parental attitudes can influence their motivation (reflective) to engage with parents *and* the social opportunity to perform the behaviors. Similarly, the emotions of embarrassment and discomfort (linked to obesity stigma) fall under automatic motivation but are also relevant to social opportunity.

**TABLE 4:** Mapping of the *barriers* to the COM‐B model

Clinical behavior	Reported barrier (sources)	COM‐B component
Using BMI to monitor weight	Lack of familiarity with using BMI^2^, ^6^, ^7^, ^13^, ^19^, ^20^, ^22^, ^25^, ^30^, ^31^, ^32^, ^38^	Psychological capability
	Uncertainty about usefulness of BMI in young children^1^, ^4^, ^13^, ^20^, ^22^, ^38^	Reflective motivation
	Time constraints^1^, ^4^, ^31^, ^32^, ^35^, ^49^	Physical opportunity
	Lack of timesaving tools (e.g., automatic BMI calculators and electronic medical records)^20^, ^49^	
Breastfeeding support	Lack of knowledge and skills^12^, ^15^, ^36^, ^39^, ^44^, ^47^ Relying on personal breastfeeding experiences as source of knowledge^15^, ^43^, ^44^, ^47^ Lack of training in breastfeeding management^15^, ^36^, ^39^, ^43^, ^44^, ^47^	Psychological capability
	Belief: breastfeeding is difficult (and formula feeding is easy)^2^, ^12^, ^28^ Attitude: prioritize supporting mother's choice^43^, ^44^ Belief: mothers lack skills and confidence^2^, ^12^, ^28^ Belief: family members and peers influence mother's infant feeding decisions^2^, ^12^, ^28^	Reflective motivation
	Time constraints^12^, ^28^, ^38^ Gap in provision of care^12^, ^43^	Social opportunity
		Physical opportunity
Providing anticipatory guidance	Deficits in knowledge about childhood obesity^6^, ^7^, ^13^, ^21^, ^22^, ^25^, ^29^, ^30^, ^32^ Lack of familiarity with guideline content^7^, ^18^, ^21^, ^22^, ^23^, ^30^, ^31^, ^48^ Lack of skills (counseling, communication)^7^, ^13^, ^18^, ^19^, ^21^, ^22^, ^23^, ^37^ Lack of obesity prevention training^2^, ^7^, ^11^, ^13^, ^18^, ^21^, ^22^, ^23^, ^29^, ^31^	Psychological capability
	Uncertainty about identifying infants at risk of developing obesity^7^, ^13^, ^14^, ^16^, ^21^, ^29^, ^30^, ^34^, ^37^, ^38^, ^46^ Disagreement with guideline content/usefulness^1^, ^8^, ^9^, ^13^, ^14^, ^19^, ^21^, ^29^, ^34^, ^37^, ^38^, ^48^, ^49^, ^50^ Attitude: prioritize family centered care over guideline implementation^13^, ^19^, ^21^, ^22^, ^29^, ^34^, ^37^ Attitude: normalize “mild” overweight^16^, ^19^, ^20^, ^21^, ^38^ Belief: PCP's prevention efforts have little impact^1^, ^2^, ^7^, ^8^, ^9^, ^10^, ^11^, ^13^, ^16^, ^18^, ^25^, ^31^, ^32^, ^40^, ^48^ Beliefs about capability: low self‐efficacy beliefs^7^, ^11^, ^13^, ^14^, ^18^, ^19^, ^21^, ^29^, ^33^, ^34^, ^38^, ^40^ Attitude: uncertainty about PCPs' role in prevention of childhood obesity^7^, ^9^, ^15^, ^18^, ^20^, ^21^, ^29^, ^34^, ^49^	Reflective motivation
	Belief: risk of harm to relationship with family^1^, ^6^, ^10^, ^11^, ^13^, ^19^, ^20^, ^21^, ^23^, ^29^, ^33^, ^34^, ^37^, ^38^, ^49^, ^50^	Social opportunity; Reflective motivation
	Beliefs, views, and assumptions about parents: Parents are resistant to advice, lack interest, not motivated^1^, ^2^, ^5^, ^6^, ^7^, ^8^, ^10^, ^11^, ^13^, ^14^, ^16^, ^18^, ^20^, ^21^, ^22^, ^30^, ^33^, ^34^, ^35^, ^37^, ^38^, ^40^, ^42^, ^48^, ^50^ Parents who are living with overweight are not concerned^6^, ^7^, ^18^, ^19^, ^21^, ^30^, ^37^, ^50^ Parents lack knowledge and skills to implement healthy weight advice^2^, ^5^, ^6^, ^13^, ^20^, ^21^, ^35^, ^37^, ^38^, ^50^ Parents' misperception of healthy child weight^2^, ^13^, ^21^, ^33^, ^34^, ^50^ Parents with socioeconomic problems are less able to implement advice^2^, ^5^, ^6^, ^7^, ^18^, ^19^, ^21^, ^23^, ^25^, ^29^, ^38^, ^50^ Influence of grandparents/peers^5^, ^13^, ^29^, ^34^ Sociocultural norms influence parental practices^2^, ^13^, ^20^, ^21^, ^33^, ^34^, ^38^, ^40^, ^50^ Parents have multiple complex health and social problems to manage^1^, ^4^, ^19^, ^21^, ^50^	
Providing anticipatory guidance	PCP's own feelings of discomfort^2^, ^5^, ^7^, ^10^, ^13^, ^19^, ^20^, ^23^, ^37^, ^38^ Fear of offending parents/parents disengaging^1^, ^5^, ^6^, ^11^, ^21^, ^29^, ^33^, ^34^, ^49^, ^50^ Previous experience of negative reactions from parents^2^, ^4^, ^13^, ^20^, ^33^, ^40^	Automatic motivation; Social opportunity
	Time constraints^1^, ^2^, ^6^, ^7^, ^8^, ^9^, ^10^, ^11^, ^13^, ^16^, ^18^, ^19^, ^21^, ^22^, ^23^, ^28^, ^30^, ^31^, ^32^, ^35^, ^37^, ^48^, ^50^ Gap in provision of care (limited opportunities for contact)^2^, ^9^, ^10^, ^20^, ^23^	Physical opportunity
	Lack of support for PCP's role from organization (budget, staffing, and resources)^7^, ^8^, ^9^, ^13^, ^18^, ^19^, ^22^, ^28^, ^36^, ^37^, ^39^	Physical opportunity
	Lack of practice tools (e.g., decision making aids and risk assessment)^1^, ^2^, ^7^, ^10^, ^13^, ^16^, ^18^, ^21^, ^22^, ^25^	Physical opportunity; Psychological capability
	Lack of support from other PCP groups in the organization^9^, ^22^, ^29^, ^37^, ^50^	Social opportunity; Reflective motivation
	Lack of a united coherent approach within the organization^4^, ^19^, ^20^, ^21^, ^37^	
	Limited access to community programs/specialists^9^, ^11^, ^13^, ^16^, ^18^, ^21^, ^22^, ^31^, ^37^	Physical opportunity; Reflective motivation

**TABLE 5:** Mapping of the *facilitators* to the COM‐B model

Clinical behavior	Reported facilitator/potential facilitator (sources)	COM‐B component
Weight assessment	Obesity training^1^, ^3^, ^4^, ^31^, ^32^ Familiarity with guidelines^31^, ^32^ Access to resources (automatic BMI calculators, support staff).^3^, ^25^, ^31^, ^32^	Psychological capability
	Belief that PCP's efforts will produce positive outcomes^1^, ^3^, ^31^, ^32^	Reflective motivation
Breastfeeding support	Knowledge and skills^36^, ^39^ Experience of working with mothers and infants^36^, ^39^ Breastfeeding training^15^, ^39^, ^41^	Psychological capability
Providing anticipatory guidance	Knowledge and confidence (self‐reported)^1^, ^5^, ^8^, ^17^, ^18^, ^22^, ^23^, ^26^, ^31^, ^32^, ^42^, ^48^ Communication skills^5^, ^8^, ^13^, ^20^, ^21^, ^22^, ^30^, ^33^, ^50^ Role specific education and training^7^, ^11^, ^22^, ^25^, ^26^, ^27^, ^35^, ^39^, ^42^ Obesity training^17^, ^31^, ^32^ Ability to use practice tools to aid communication.^4,5,13,20–22,30,33,504,13,20,21,33,50^ Experience of working with children and mothers^2^, ^4^, ^29^, ^36^, ^39^ Access to training opportunities^2^, ^4^, ^17^, ^31^, ^32^, ^42^, ^43^, ^48^	Psychological capability
	Positive attitudes and intention^5^, ^7^, ^8^, ^18^, ^20^, ^21^, ^30^, ^31^, ^32^, ^43^, ^47^, ^49^ Expectations of positive outcomes of PCP's prevention efforts.^3^, ^17^, ^31^, ^32^	Reflective motivation
	Positive relationship with family^9,10,13,21,33,49,10,12,33,49^ Parental concern about child overweight^3^, ^20^	Social opportunity
	Perception of support from organization for PCP's role^4^, ^8^, ^11^, ^18^, ^32^	Reflective motivation
	Availability of sufficient time and support staff^12^, ^13^, ^23^, ^31^	Physical opportunity
Providing anticipatory guidance	Access to specialist staff and community based programs^13^, ^17^, ^42^	Physical opportunity; Reflective motivation
	Uniform coherent approach within the organization^8^, ^9^, ^20^, ^50^ Closer working between doctors and nurses^4^, ^20^, ^43^, ^50^	Social opportunity; Reflective motivation

The analyses suggested that various factors influence PCPs' motivations to implement childhood obesity prevention practices. PCPs who lacked knowledge and skills (psychological capability) and perceived resistance from parents (social opportunity) were less likely to perform the behaviors (e.g., raise the topic of weight) and more likely to report low expectations of positive outcomes of their prevention efforts (reflective motivation). In contrast, PCPs who believed they were competent (psychological capacity) and experienced role support from the provider organization (physical and social opportunity) were motivated to perform the behaviors. Further, the analyses suggest that engaging in a behavior that requires skill can improve capability; PCPs whose role required them to frequently provide infant feeding advice to parents reported higher levels of confidence in performing this task (psychological capability) than PCPs who had simply completed role‐specific specialist training. These findings reflect the hypothesized linkages between the subcomponents of the COM‐B.^22^

## 4 DISCUSSION

Building on previous work in this area,^75^, ^76^ this review confirmed that PCPs inconsistently comply with recommended practices and perceive various barriers to implementation of guidelines. These barriers influence their capability, opportunity, and motivation to perform the recommended practices. There was a high degree of consistency of the findings across the 50 studies that originated from different countries, with no significant differences between PCPs from different professions with regard to the barriers and facilitators. The evidence synthesized from the additional five studies included in the review update did not generate new concepts or add depth to concepts that were already identified in the evidence synthesized from the 45 studies identified from searches carried out in March 2018. Implementation differed in terms of PCPs' views about the recommended behaviors and their beliefs about the time and skills required in delivering them. PCPs who were specifically trained to address childhood obesity and worked in a supportive practice environment were more likely to implement guidelines. A trusting PCP‐parent relationship was described as a key facilitator; however, the value attached to maintaining the relationship acted as a barrier. The review also identified communication strategies used by PCPs to overcome barriers, PCPs' resource and training needs and their recommendations to improve the delivery and quality of services.

It must be acknowledged that the data related to barriers and facilitators are attributions that PCPs make about their own behaviors, not the actual determinants of their practices. Barriers to change are socially constructed by practitioners to justify the situation they are in and preserve their social and professional identity.^77^ PCPs attributed their lack of skill and confidence to a lack of training and identified many barriers external to them. PCPs' belief that parents and organizations are lacking in their efforts may have contributed to their sense of futility with regard to the potential impact of obesity prevention efforts.

Due to the potential of this attributional bias, caution must be exercised when interpreting the findings of the barriers and facilitators.

### 4.1 Gaps in literature

Several gaps emerged from the data. Firstly, there was lack of information on collaborative working and team‐based approach to implementation of guidelines. Much of the data presented in this review focuses on the individual PCP's practices and their attributes. Second, the lack of time was a frequently reported barrier; however, there was little data on how PCPs managed competing priorities during time‐constrained consultations with families. Thirdly, there was limited data on the relative importance of different contextual factors and how these may have influenced each other and practice behaviors. This may be because the research methodologies (e.g., qualitative longitudinal case study design) required to capture the complexity and dynamic nature of context and its impact on implementation are typically resource intensive.^78^

### 4.2 Implications for policy and practice

There are missed opportunities in primary care for addressing prevention of overweight in young children. All PCP groups expressed the need for training and resources, suggesting that PCPs believed that they should address the issue. Improving adherence to recommended practices will likely require a range of professional development activities focused on building PCPs' capability, attitudes, and self‐efficacy beliefs, and also shifting their views about the importance and impact of early prevention interventions. Furthermore, embedding guidelines into PCPs' existing routines will require support for the PCP's role, such as clear care pathways, decision support tools, and access to training and referral services. Similar findings have been reported by previous research on this topic.^79^, ^80^ Implementation will likely require policies to support service delivery models that focus on early intervention, promote a collaborative approach between different PCP groups, offer continuity of care, and address PCPs' case workload issues.

### 4.3 Strengths and limitations

To our knowledge, this is the first systematic review to report on childhood‐obesity prevention practice behaviors of key PCP groups and conduct a theoretical analysis of the barriers and facilitators. Updating of the searches of the databases in April 2021 provided reassurance that there were no emerging issues. The inclusion of studies of diverse research designs, involving all key PCP groups and different organizational and social contexts, ensures a rich and comprehensive dataset. The application of an aggregated model of behavior (COM‐B model) has helped with developing an understanding of how different factors influence PCP's performance of the recommended practice behaviors.

Several limitations of this research must be acknowledged. Given the countries of origin of the included studies, the findings of this review are likely to be relevant only to high‐income countries. Limiting the searches to English language publications may have excluded relevant studies from countries with different socioeconomic and cultural profile that may have very different experiences and needs. All studies that met the inclusion criteria were included irrespective of the assessment of their quality; this may have affected the quality of the data that was synthesized. The data presented is subject to different sources of bias, notably selection bias and social desirability bias.

This review was led by DR as part of a doctoral research project and is “restricted” because certain elements that are required in a “full” review were simplified.^81^ A single reviewer screening of abstracts may limit the methodological standard of a review.^82^ However, the conduct of the review was closely supervised by DR's supervisors who were also members of the review team. As second reviewers, review team members verified the lead reviewer's work on quality assessment and data extraction on a randomly selected proportion of the papers. A well conducted restricted review with minimum 20% checking by a second reviewer is considered an appropriate strategy in situations where a “full” SR process cannot be implemented.^82^, ^83^

## 5 CONCLUSION

The review has highlighted the challenges associated with implementing guidelines for prevention of obesity in 0–5 year old children. Application of a theoretical framework to the synthesis of the data has provided insights into the interacting processes by which practitioners' knowledge, beliefs, and attitudes influence implementation. This research was the first step toward developing an intervention to strengthen health visitors' role in prevention of excess weight gain in 0–2 year olds in an area in Northeast England. The review identified important gaps in the literature. Studies are required beyond identifying the barriers and facilitators; these will need a more explanatory and theory‐driven approach to investigate how and why “barriers to change” influence implementation. Another area for future work is exploration of how and why specific contextual factors influence implementation, their relative importance, and interactions between them.

## CONFLICT OF INTEREST

There are no other relationships/conditions/circumstances that present a potential conflict of interest.

## Supporting information


**Table S1.** Reporting of the Systematic Review using the PRISMA 2020 checklist.
**Figure S1.** MEDLINE Search strategy
**Table S2**. Characteristics of included studies are listed in the order of their publication date, from most recent to oldest.
**Table S3.** Overview of the barriers with indicative quotes.
**Table S4.** Overview of the facilitators with indicative quotes.
