# Supplementary material for: Barriers and facilitators to implementing practices for prevention of childhood obesity in primary care: A mixed methods systematic review
Source: Obes Rev. 2022 Jan 22;23(4):e13417. doi: 10.1111/obr.13417 (PMC9285925; doi:10.1111/obr.13417)
Supplement: Supplementary file 1 — Table S1. Reporting of the Systematic Review using the PRISMA 2020 checklist. Figure S1. MEDLINE Search strategy Table S2. Characteristics of included studies are listed in the order of their publication date, from most recent to oldest. Table S3. Overview of the barriers with indicative quotes. Table S4. Overview of the facilitators with indicative quotes. [file OBR-23-0-s001.docx]

**Supporting information**

**Article title:** Barriers and facilitators to implementing practices for prevention of childhood obesity in primary care: a mixed methods systematic review.

Authors’ names: Devashish Ray^1^, Falko Sniehotta^1^, Elaine McColl^1^, Louisa Ells^2^

^1^Population Health Sciences Institute, Newcastle University; ^2^ School of Clinical and Applied Sciences, Leeds Beckett University

Address of corresponding author and email address:

Devashish Ray, Population Health Sciences Institute, Newcastle University, Newcastle-upon-Tyne, NE2 4AX, U.K. Email: d.ray2@newcastle.ac.uk

**Table S1. Reporting of the Systematic Review using the PRISMA 2020 checklist**.

[Abbreviations: PROSPERO= Prospective Register of Systematic Reviews; JBI = Joanna Briggs Institute]

| **Section and Topic** | **Item #** | **Checklist item** | Where reported |
| --- | --- | --- | --- |
| **TITLE** | | |  |
| Title | 1 | Identify the report as a systematic review. | Title page |
| **ABSTRACT** | | |  |
| Abstract | 2 | \| A summary is included; the structure of the summary follows the guidance for authors published by this journal \| \| --- \| | Yes; “Abstract” submitted separately |
| **INTRODUCTION** | | |  |
| Rationale | 3 | Describe the rationale for the review in the context of existing knowledge. | 1-2 |
| Objectives | 4 | Provide an explicit statement of the objective(s) or question(s) the review addresses. | 2 |
| **METHODS** | | |  |
| Eligibility criteria | 5 | Specify the inclusion and exclusion criteria for the review and how studies were grouped for the syntheses. | 3; Table 1 |
| Information sources | 6 | Specify all databases, registers, websites, organisations, reference lists and other sources searched or consulted to identify studies. Specify the date when each source was last searched or consulted. | 4 |
| Search strategy | 7 | Present the full search strategies for all databases, registers, and websites, including any filters and limits used. | 4; Figure S1 |
| Selection process | 8 | Specify the methods used to decide whether a study met the inclusion criteria of the review, including how many reviewers screened each record and each report retrieved, whether they worked independently, and if applicable, details of automation tools used in the process. | 4 |
| Data collection process | 9 | Specify the methods used to collect data from reports, including how many reviewers collected data from each report, whether they worked independently, any processes for obtaining or confirming data from study investigators, and if applicable, details of automation tools used in the process. | 5 |
| Data items | 10a | List and define all outcomes for which data were sought. Specify whether all results that were compatible with each outcome domain in each study were sought (e.g. for all measures, time points, analyses), and if not, the methods used to decide which results to collect. | 3, 5 |
|  | 10b | List and define all other variables for which data were sought (e.g. participant and intervention characteristics, funding sources). Describe any assumptions made about any missing or unclear information. | 3, 5 |
| Study risk of bias assessment | 11 | Specify the methods used to assess risk of bias in the included studies, including details of the tool(s) used, how many reviewers assessed each study and whether they worked independently, and if applicable, details of automation tools used in the process. | 4-5 |
| Effect measures | 12 | Specify for each outcome the effect measure(s) (e.g. risk ratio, mean difference) used in the synthesis or presentation of results. | Not applicable |
| Synthesis methods | 13a | Describe the processes used to decide which studies were eligible for each synthesis (e.g. tabulating the study intervention characteristics and comparing against the planned groups for each synthesis (item #5)). | Not applicable |
|  | 13b | Describe any methods required to prepare the data for presentation or synthesis, such as handling of missing summary statistics, or data conversions. | Not applicable |
|  | 13c | Describe any methods used to tabulate or visually display results of individual studies and syntheses. | 6-7 |
|  | 13d | Describe any methods used to synthesize results and provide a rationale for the choice(s). If meta-analysis was performed, describe the model(s), method(s) to identify the presence and extent of statistical heterogeneity, and software package(s) used. | 5-6 |
|  | 13e | Describe any methods used to explore possible causes of heterogeneity among study results (e.g. subgroup analysis, meta-regression). | Not applicable |
|  | 13f | Describe any sensitivity analyses conducted to assess robustness of the synthesized results. | Not applicable |
| Reporting bias assessment | 14 | Describe any methods used to assess risk of bias due to missing results in a synthesis (arising from reporting biases) | Not applicable |
| Certainty assessment | 15 | Describe any methods used to assess certainty (or confidence) in the body of evidence for an outcome | Not reported |
| **RESULTS** | | |  |
| Study selection | 16a | Describe the results of the search and selection process, from the number of records identified in the search to the number of studies included in the review, ideally using a flow diagram | 4, 6; Figure 1 |
|  | 16b | Cite studies that might appear to meet the inclusion criteria, but which were excluded, and explain why they were excluded. | Figure 1 |
| Study characteristics | 17 | Cite each included study and present its characteristics. | Table S4 |
| Risk of bias in studies | 18 | Present assessments of risk of bias for each included study | Available from first author upon request (page 7) |
|  |  | Risk of bias across the different studies | Tables S2 and S3 |
| Results of individual studies | 19 | For all outcomes, present, for each study: (a) summary statistics for each group and (b) an effect estimate and its precision (e.g. confidence/credible interval), ideally using structured tables or plots. | Not applicable |
| Results of syntheses | 20a | For each synthesis, briefly summarise the characteristics and risk of bias among contributing studies | 7 |
|  | 20b | Present results of all statistical syntheses conducted. If meta-analysis was done, present for each the summary estimate and its precision (e.g. confidence/credible interval) | Not applicable; |
|  | 20c | Present results of all investigations | 7-15 (narrative synthesis) |
|  | 20d | Present results of all sensitivity analyses conducted to assess the robustness of the synthesized results | Not applicable |
|  |  | Present results of additional analysis | 16 |
| Reporting biases | 21 | Present assessments of risk of bias due to missing results (arising from reporting biases) for each synthesis assessed | Not applicable |
| Certainty of evidence | 22 | Present assessments of certainty (or confidence) in the body of evidence for each outcome assessed. | Not presented |
| **DISCUSSION** | | |  |
| Discussion | 23a | Provide a general interpretation of the results in the context of other evidence. | 17-18 |
|  | 23b | Discuss any limitations of the evidence included in the review. | 18 |
|  | 23c | Discuss any limitations of the review processes used. | 19-20 |
|  | 23d | Discuss implications of the results for practice, policy, and future research. | 18-20 |
| **OTHER INFORMATION** | | |  |
| Registration and protocol | 24a | Provide registration information for the review, including register name and registration number, or state that the review was not registered. | PROSPERO; CRD42017084067 |
|  | 24b | Indicate where the review protocol can be accessed, or state that a protocol was not prepared. | Accessible on PROSPERO |
|  | 24c | Describe and explain any amendments to information provided at registration or in the protocol. | PROSPERO protocol registration page |
| Support | 25 | Describe sources of financial or non-financial support for the review, and the role of the funders or sponsors in the review. | Title page |
| Competing interests | 26 | Declare any competing interests of review authors. | Authors’ Disclosure forms |
| Availability of data, code and other materials | 27 | Report which of the following are publicly available and where they can be found: template data collection forms; data extracted from included studies; data used for all analyses; analytic code; any other materials used in the review. | Data extraction forms available from JBI website; all other data available from first author upon request |

**Figure S1. MEDLINE Search strategy**

| 1. (Child* adj2 (Obesity or overweight)).mp. |  |
| --- | --- |
| 2. ((infant or toddler or baby) adj2 (obesity or overweight or excess weight)).mp. |  |
| 3. (bodyweight or body weight or body-weight).ab,ti. |  |
| 4. ((child* adj3 body mass index) or bmi).ab,ti. |  |
| 5. (weight adj3 (gain or maintenance or management)).ab,ti. |  |
| 6. ((prevent* or manage) adj3 (childhood obesity or obesity in children or childhood overweight or overweight in children)).ab,ti. |  |
| 7. ((Guidance* or guideline* or recommended or recommendation* or advice or advised or standard$ or statement or consensus or policy or policies or protocol*) adj10 (implement* or aware* or uptake or up-take or takeup or take-up or adhere or adherence or concordance or accordance or fidelity or adopt* or comply or compliance)).mp. |  |
| 8. (barrier* or difficulty or difficulties or issues or challenges or facilitat* or enablers or motivators).ab,ti. |  |
| 9. "Attitude of Health Personnel"/ or Health Knowledge, Attitudes, Practice/ or "Surveys and Questionnaires"/ or Health Personnel/ |  |
| 10. Communication/ or Professional-Family Relations/ or parent-nurse communication.mp. |  |
| 11. (perception* or opinion* or experience* or insight* or understand* or belief* or knowledge or behavio?r or role* or view or view-point or point of view or role*).ab,ti. |  |
| 12. Quality Improvement/ or Professional Practice/ or "Attitude of Health Personnel"/ or Primary Health Care/ or Obesity/ or Health Personnel/ |  |
| 13. (behaviour* or behavior* or behaviour* change or behaviour* change).ab,ti. |  |
| 14. (weight monitoring adj10 (child* or infant or baby or babies or toddler*)).ab,ti. |  |
| 15. responsive feeding.ab,ti. |  |
| 16. ((advice or counsel) adj5 breastfeeding).ab,ti. |  |
| 17. ((discuss or advice) adj10 (bottlefeeding or bottle-feeding or formula feeding)).mp. |  |
| 18. ((discuss* or talk* or "rais* the topic" or advice) adj10 (infant weight or child weight or toddler weight)).ab,ti. |  |
| 19. ((discuss* or talk* or advice*) adj10 (infant sleep or baby* sleep or toddler sleep)).ab,ti. |  |
| 20. ((discuss* or talk* or advice*) adj10 (physical activity or play)).ab,ti. |  |
| 21. ((discuss* or talk* or advice*) adj10 (weaning or complementary feeding)).ab,ti. |  |
| 22. ((discuss* or advice*) adj10 (healthy diet or nutrition)).ab,ti. |  |
| 23. ((discuss* or talk* or advice*) adj10 portion size*).ab,ti. |  |
| 24. (healthcare professional or health care professional or health professional).ab,ti. |  |
| 25. ((child health care or nurse or family or general) adj3 (provider or practitioner*)).ab,ti. |  |
| 26. (("public health" or "community health" or "community") adj3 nurse*).ab,ti. |  |
| 27. (doctor or p$ediatric*).ab,ti. |  |
| 28. (child health service* or child* health cent$r* or well-baby clinic or primary care or community cent$r* or community health cent$r*).ab,ti. |  |
| 29. ((home or health) adj3 visitor).ab,ti. |  |
| 30. 1 or 2 or 3 or 4 or 5 or 6 |  |
| 31. 14 or 15 or 16 or 17 or 18 or 19 or 20 or 21 or 22 or 23 |  |
| 32. 7 and 8 |  |
| 33. 9 or 10 or 11 or 12 or 13 |  |
| 34. 31 or 32 or 33 |  |
| 35. 24 or 25 or 26 or 27 or 28 or 29 |  |
| 36. 30 and 34 and 35 |  |
| 37. limit 36 to (english language and humans and yr="2002 -Current" and ("newborn infant (birth to 1 month)" or "infant (1 to 23 months)" or "preschool child (2 to 5 years)")) |  |

**Table S2**. Characteristics of included studies are listed in the order of their publication date, from most recent to oldest.

Abbreviations: *GP* general practitioner; *HV* Health Visitor; *NP* Nurse practitioners; *PED* Paediatrician; *PNP* Paediatric nurse practitioner; *PA* physician assistant;

*WIC* Women, infants. and children; *FP* Family physician; *FNP* Family nurse practitioner; *RN* Registered nurse; *BF* breastfeeding; AAP= American Academy of Pediatrics

| **First author; Year** | **Country** | **Participants characteristics; sample size** | **Key aims/ objectives of study** | **Service-user group/ topic** | **Study design and data collection methods** | **Primary care setting/context** |
| --- | --- | --- | --- | --- | --- | --- |
| Andersen; 2020 [^1^](#_ENREF_1) | USA | PEDs, NPs, PNPs; PAs; n=20 | Explore PCPs’ perspectives on evaluating and communicating about early excessive weight gain and to identify PCP-opined barriers | 0-5 years | Qualitative; semi-structured interview | Community, academic or hospital affiliated, and multi-speciality affiliated clinics |
| Cheng, 2020 [^2^](#_ENREF_2) | Australia | Child and Family Health nurses (CFHNs); survey, n = 90; interview, n=20 | Examine factors influencing the practices of CFHNs to prevent childhood obesity | 0-5 years | Mixed: qualitative and quantitative; Survey questionnaire and interviews | Maternal and child health service government clinics |
| Belay; 2019 [^3^](#_ENREF_3) | USA | PEDs; Total sample from 3 surveys; n=1805 | Compare PEDs’ practices and attitudes regarding BMI assessment and counselling in children ≥ 2 years in 2006, 2010 and 2017 | 2-17 years | Quantitative; routine national surveys of AAP members | Nation-wide survey of AAP members who work in well-child clinics |
| Moir; 2019 [^4^](#_ENREF_4) | New Zealand | Maternal and Child Health (MCH) nurses; n=33 | Experiences of conducting the mandatory BMI assessment as part of routine before school health check | 4 year olds | Qualitative; Focus groups | Pre-school routine health check clinic (national government programme) |
| Kracht; 2019 [^5^](#_ENREF_5) | USA | Nurses, physicians, dietitians); n=20 | Develop an understanding of how PCPs perceive their role in healthy development and prevention of obesity in young American Indian (AI) children | 0-5 years | Qualitative; semi-structured interviews | Primary care clinics (including government clinics) serving AI families |
| Tanda; 2017 [^6^](#_ENREF_6) | USA | Nurse practitioners (NPs); n=155; | Practitioners’ knowledge and practice patterns; perceived barriers to implement recommended practices | 2-17 years | Quantitative; Survey questionnaire (with open ended questions) | Clinics included physician-owned (22%), hospital affiliated (33.5%), large medical groups (31%) |
| Dera-de Bie; 2016 [^7^](#_ENREF_7) | Netherlands | GPs and Child Health Centre (CHC) nurses (n=216) | Identify PCPs’ behavioural and personal characteristics that influence their practices for prevention of childhood obesity | 0-4 years | Quantitative; Survey questionnaire | Child health centres (national level programme) |
| Nordstrand; 2016 [^8^](#_ENREF_8) | Norway | Public Health Nurses (PHNs); n=18. | Determine implementation of guidelines for prevention of childhood obesity | 0-5 years | Qualitative; semi-structured interview | Well-baby clinics (national government programme) |
| Ditlevsen; 2016 [^9^](#_ENREF_9) | Denmark | GPs and Health Visitors (HVs); n=19 (10 GPs and 9 HVs) | Identify problems experienced by GPs and HVs that influence implementation of recommended practices | 3-5 years | Qualitative; semi-structured interview | Services delivered by GPs and public health services, local authority |
| Bourgeois; 2016 [^10^](#_ENREF_10) | Canada | Clinicians and nurses; n= 40 | Determine PCPs’ perceptions of facilitators of and barriers to prevention of childhood obesity | 2-5 years | Qualitative; Focus groups | Family Health Teams: 2 urban practices (1 academic, 1 community-based); 1 rural practice |
| Nelson;  2015 [^11^](#_ENREF_11) | USA | PEDs; FPs; NPs/ PAs; (265 PEDs, 143 FPs, 248 NP/ PAs); n=656 | Assess PCPs’ perceptions and weight management practices; perception of top 3 barriers and top 3 training/ resource needs | Included 0-5-year | Quantitative; Survey questionnaire | Well-child clinics (State-wide survey study) |
| McLelland; 2015 [^12^](#_ENREF_12) | Australia | MCH nurses; n=17 (12 nurses, 5 domiciliary midwives) | Explore views regarding factors that influence breast feeding initiation and continuation | Breastfeeding  support | Qualitative; Focus groups | Community based home visiting services (local government funded) |
| Laws; 2015 [^13^](#_ENREF_13) | Australia | MCH nurses; survey, n=56; interviews, n= 16 | Examine practices for prevention of obesity in early life; factors influencing the practices | 0-5 years | Mixed: Survey questionnaire and interviews | Maternal and child health services (local government funded) |
| Chelva-kumar; 2014; [^14^](#_ENREF_14) | USA | Physicians; PAs; NPs; n=69 | Assess providers’ perception and documentation of their adherence to guidelines | 2-18 years | Quantitative: survey questionnaire; retrospective chart review | Paediatric primary care clinics in a large urban city |
| Pound; 2014; [^15^](#_ENREF_15) | Canada | Doctors; n= 780; (397 PEDs; 322 FPs; 61 resident doctors); | Assess breastfeeding knowledge**,** confidence, beliefs, and attitudes | Breastfeeding  support | Quantitative; Survey questionnaire | Nation-wide survey of Pediatricians and Family physicians |
| Bonnet; 2014; [^16^](#_ENREF_16) | USA | Physicians, PAs, NPs; n=56 (41 physicians, 8 PAs & 7 NPs) | Examine perceived barriers for prevention of childhood obesity and practice related behaviours | 0-5 years | Quantitative; Survey questionnaire | Family medicine clinics affiliated to University network |
| Lowenstein; 2013; [^17^](#_ENREF_17) | USA | Physicians; NPs; PAs; n=123 | Explore the relationship of providers’ self-efficacy, outcome expectations,  and practice characteristics | 3-8 years | Quantitative; Survey questionnaire | Paediatric & family medicine practices, community centres in rural & suburban areas |
| Robinson; 2013; [^18^](#_ENREF_18) | Australia | General practice nurses; survey, n=59; interviews, n=10 | Explore current practice, attitudes, confidence, and trainings needs of nurses | Child’s 4-year review | Mixed: quantitative and qualitative; Survey  questionnaire and interviews | General practices (government programme) |
| Ljungkrona-Falk; 2013; [^19^](#_ENREF_19) | Sweden | Child health care centre nurses; surveys, n= 62; three focus groups, n= 17) | Explore perceived barriers when discussing with parents food habits, physical activity, and their child’s body weight | 18 months- 3 years | Mixed: qualitative and quantitative; Focus groups and survey questionnaire | Child Health care centres (local government funded) |
| Regber; 2013;  [^20^](#_ENREF_20) | Sweden | Child health care centre nurses; n=15 | Examine nurses’ practice related behaviours to promote healthy weight gain in children and prevention of childhood obesity | 0-6 years | Qualitative; Semi-structured interviews | Children health care centres (local government) |
| Redsell;  2013; [^21^](#_ENREF_21) | UK (England) | Nurses; n=23 (20 HVs, 3 RNs, 7 neonatal nurses) | Explore PCPs’ beliefs and current practices in identifying and intervening with infants at risk of developing obesity | 0-2 years | Qualitative; Semi-structured interviews | Primary care trusts (England NHS) |
| Isma; 2013; [^22^](#_ENREF_22) | Sweden | Child Health centre nurses; n=18 | Explore nurses’ concepts of their practices related to prevention of childhood obesity | 0-6 years | Qualitative; Open-ended interviews | Primary care child health services (local government) |
| Findholt; 2013; [^23^](#_ENREF_23) | USA | Family medicine physicians (FMPs); PEDs; PAs; NPs; n=13 | Explore PCPs’ perceived barriers, resources, and training needs | 0-18 years | Qualitative; In-depth interviews | Pediatric & family practices in rural counties of a US State |
| Bohman; 2013; [^24^](#_ENREF_24) | Sweden | Child health nurses; n=23 | Investigate the extent to which conversations between nurses and parents focus on child dietary and physical activity behaviours | 2.5 - 4 years | Qualitative; Recordings of conversations between nurse and parent at a well-child visit | Primary care child health services (local government) |
| Dera-de bie; 2012; [^25^](#_ENREF_25) | Netherlands | Child health practitioners; n=12 (6 physicians and 6 nurses) | Explore perceptions of PCPs on prevention of overweight in infants in routine practice | 0-1 year | Qualitative; in-depth Interviews | Child healthcare services (national government programme) |
| Isma; 2012;  [^26^](#_ENREF_26) | Sweden | Child Health nurses; n=18 | Explore nurses’ perspectives of prevention of childhood overweight and obesity | 0-6 years | Qualitative; Open-ended interviews | Primary care child health services (local government) |
| Rausch; 2011; [^27^](#_ENREF_27) | USA | FPs and PEDs; n=96 (PEDs, 81%; Family medicine, 14.6%) | Explore attitudes and practices recommended for prevention of childhood obesity | 2-18 years | Quantitative; Survey questionnaire (with open-ended questions | Community based, hospital-affiliated practices |
| Wethington; 2011; [^28^](#_ENREF_28) | USA | GPs and PEDs; n=871 (250 PEDs and 621 GPs) | Determine the proportion of GPs and PEDs who follow recommendations on prevention of childhood obesity | 2- 19 years | Quantitative; Secondary analyses of data collected from a large survey study | Secondary analysis of national survey of primary care providers |
| Huang; 2011; USA [^29^](#_ENREF_29) | USA | Family practice physicians & PEDs; n= 811 | Explore assessment, counselling, and routine practices for management of diet, physical activity, and weight status among paediatric patients | 0-2 &  2-17 years | Quantitative; Survey questionnaire (included two different case vignettes) | Study was part a nation-wide survey of primary care providers |
| Brown; 2011; [^30^](#_ENREF_30) | UK (England) | HVs; midwives; n=20 (midwives 4, HVs 4, BF counsellors 4, others 8); | Explore PCPs’ perceptions of factors that influence mother’s infant feeding decisions; explore PCPs’ perceived barriers | Breastfeeding  support | Qualitative; Semi-structured Interviews | Community health services, English NHS |
| Redsell;  2011; [^31^](#_ENREF_31) | UK (England) | GPs, practice nurses, HVs; n=118 (survey); n=18 (interviews) | Explore knowledge of obesity; beliefs and current practices related to preventing childhood obesity during infancy | 0-1 year | Mixed: quantitative and qualitative; Survey questionnaire and interviews | NHS primary care trusts and community health services in England |
| Spivack; 2010; [^32^](#_ENREF_32) | USA | PEDs and NPs; n=87 (80 PEDs and 7 NPs) | Evaluate knowledge, current practices, and perceived barriers to prevent development of obesity in children at first year visit | 1 year well-child visit | Quantitative; Survey questionnaire | First-year well-childcare visits in primary care practices |
| Sesselberg;  2010; [^33^](#_ENREF_33) | USA | Family physicians; n=445 | Examine PCPs’ attitudes and practices about prevention of obesity, screening and use of body mass index (BMI) percentiles | 0-18 years | Quantitative; Survey questionnaire | Clinics run by family physicians |
| Klein; 2010; [^34^](#_ENREF_34) | USA | PEDs; n=677 | Examine PEDs’ implementation of guideline based screening including use of BMI percentile | 0-18 years | Quantitative; Survey questionnaire | Rural (15%), suburban (44%) and urban (41%) primary care facilities |
| Edvardsson; 2009; [^35^](#_ENREF_35) | Australia | MCH nurses; n=10 | Explore nurses’ experiences of raising issues related to prevention of overweight in children with parents | 0-5 years | Qualitative; Open-ended interviews | Culturally diverse rural and urban areas |
| Johnson; 2008; [^36^](#_ENREF_36) | USA | Primary care providers (government funded); n=38 | Examine PCPs’ perceptions of the feeding practices & behaviours, and cultural variables thought to contribute to development of obesity in infants | Infants | Qualitative; Focus groups (five in all, each with a homogenous group) | Mexican American communities in an urban area |
| Woolford; 2008; [^37^](#_ENREF_37) | USA | PEDs and FPs; n= 267 | Explore barriers and facilitators to using BMI for pre-schoolers | 0-5 years | Quantitative; Survey questionnaire | Primary care well-child practices affiliated to a local hospital network |
| Wallace; 2007; [^38^](#_ENREF_38) | UK (England) | HVs & midwives; n=549 (HVs, 33%; midwives, 37%; breastfeeding support staff); | Assess perceptions of competence and training needs, organisational barriers, and preferences with regard to breastfeeding support to mothers | Breastfeeding  support | Quantitative; Survey questionnaires | National Learning Needs Assessment study (national survey) |
| Wallace; 2006; [^39^](#_ENREF_39) | UK (England) | PEDs and GPs; n= 177 (GPs=57; PEDs=120) | Examine perceptions of competence, skills, knowledge of policies on breastfeeding; training needs; organisational barriers | Breastfeeding  support | Quantitative; Survey questionnaire | National Learning Needs Assessment study (national survey) |
| Serrano; 2006; [^40^](#_ENREF_40) | USA | WIC staff (nurses, nutritionists, nutrition assistants); n= 64 | Assess attitudes, perceptions, and practices of staff in addressing development of overweight in children | 0- 5 years | Quantitative; Survey questionnaire (included open -ended questions) | National public funded program for mothers and 0-5-year-olds |
| Tappin; 2006; [^41^](#_ENREF_41) | UK (Scotland) | Health Visitors; n=146 | Document individual HV role: interventions, activities, and attitude towards breastfeeding. | Breast-feeding support | Quantitative; Survey questionnaire; routinely collected practice data | UK Health visiting (NHS Scotland) |
| Larsen; 2006; [^42^](#_ENREF_42) | USA | Family NPs and Paediatric NPs; n=99 | Describe NPs’ childhood obesity prevention practices; examine barriers & facilitators; compare practice across setting and specialty | 0-5 years | Quantitative; Survey questionnaire (included open-ended questions) | Family practice and general paediatric practice; USA State-wide survey study |
| Tennant; 2006; [^43^](#_ENREF_43) | UK (England) | HVs and midwives; n=10 | Explore the perceptions of their skills and attitudes to supporting breastfeeding mothers, to examine barriers and drivers for change | Breast- feeding support | Qualitative; Focus groups | Primary care trust (NHS England community services) |
| Smale; 2006; [^44^](#_ENREF_44) | UK(England) | Primary care staff; n=73 (14 midwives, 19 HVs, 4 GPs, 3 PEDs, 2 nurses) | Explore views and perspectives of NHS staff, with the aim to identify breastfeeding training needs analysis | Breast-feeding support | Qualitative; individual interviews and group interviews | Primary care trusts; NHS England community services |
| Perrin; 2004;  [^45^](#_ENREF_45) | USA | Paediatricians; n=356 | Determine paediatricians’ use of BMI to assess growth of children | Including 0-5-year-olds | Quantitative; Survey | Wide variety of practice settings; a US State-wide survey study |
| Rattay; 2004; USA [^46^](#_ENREF_46) | USA | PEDs who saw ≥ ten 2-18-year-olds/ week; n= 813 | Explore practice related to counselling children about healthy weight/weight related topics | 2-18 years | Quantitative; Survey questionnaire | Variety of paediatric primary care practices: nation-wide survey |
| Hellings; 2004; [^47^](#_ENREF_47) | USA | Paediatric nurse practitioners (PNPs); n=77 | Examine PNPs’ knowledge, attitudes of breastfeeding and management of breastfeeding problems | Breast-feeding care | Quantitative; secondary analyses of data from a previous larger study | Secondary analysis of data from a previous US state-wide survey |
| Gentile; 2004; [^48^](#_ENREF_48) | USA | PEDs; n=365 | Assess awareness of, agreement with, and implementation of recommendations to limit children’s TV viewing/media time | 0-2 years | Quantitative; Survey questionnaire | Paediatricians registered with the AAP chapter of one US State |
| Gilbert; 2004; [^49^](#_ENREF_49) | USA | PEDS; n=24 | Explore PEDs’ attitudes to their role in providing anticipatory guidance to prevent development of obesity in children | 0-17 years | Qualitative; Open-ended Interviews | Pediatric primary care: community practice or academic settings |
| Chamberlin; 2002; [^50^](#_ENREF_50) | USA | WIC staff; n=19 (7 clinical nutritionists; 12 nurses | Examine perceptions of staff about challenges with prevention of development of obesity in 0-5 year old children | Pre-school children | Qualitative; Focus groups and individual interviews | Government funded program for mothers and 0-5-year-olds |

**Table S3. Overview of the barriers with indicative quotes.**

[Abbreviations: BF=breastfeeding; BMI=body mass index; HCP= healthcare practitioner; HV= Health Visitor; PCP=primary care practitioner]

| Category | Finding | Indicative quotes |
| --- | --- | --- |
| Barriers to use  of BMI: | Lack of knowledge and skills | *“No, I haven’t calculated BMI, partly because I don’t master it well… and partly because I don’t know the threshold values…I could have looked it up but then I don’t feel comfortable about informing the parents”* (Nurse)[^22^](#_ENREF_22) |
|  | Uncertainty about usefulness of BMI | *“I don’t look at them (growth or BMI charts) all that much because it doesn’t take into effect their race, the parents’ size or anything like that”* (Nurse)[^13^](#_ENREF_13)    *“… I do not agree with what I see in front of me. The curves in the chart can show that the children are proportionate, but the BMI shows that they are over the threshold values”* (Nurse)[^22^](#_ENREF_22) |
|  | Time constraints | *“I can offer to take an extra weight measurement and such, but I have neither the time nor the knowledge to do so very much, really”* (Nurse)[^22^](#_ENREF_22) |
| Barriers to providing breastfeeding support and  care  Barriers to providing breastfeeding  support and  care | Deficit in BF knowledge | *“The simple things need to be understood, the more you do the more you realise you do not know. (I) feel it is outrageous and scary that paediatricians do not get specific training in breastfeeding...”* (Physician)[^44^](#_ENREF_44) |
|  | Using personal BF experiences as source of knowledge | *“I had all these awful emotions that women have when breastfeeding doesn't work, so from a positive point of view I feel I can support women with that, but that's because of using my own experience and putting it into a professional context. But I could see how easily someone could say ‘well, I had to give up’ and it can go the other way”* (HV)[^43^](#_ENREF_43) |
|  | Lack of skills | *“I don’t think that they (GPs) are ignorant about the benefits of it [breastfeeding], but don’t know the process and how to promote it…”* (Nurse)[^12^](#_ENREF_12) |
|  | Belief: BF is difficult for mothers | *“A lot of mums just don’t realise the reality of having a new-born baby. I mean, it’s exhausting and it’s no wonder they chose what appears to be the easiest method. Unfortunately, formula does seem to settle babies more quickly even though that’s not necessarily good for them”* (PCP)[^30^](#_ENREF_30) |
|  | Prioritise supporting mother’s choice | *“Ultimately you've got to support the woman in her decision, whatever she wants to do and I think that's very important.”* (Nurse)[^43^](#_ENREF_43) |
|  | Mothers lack knowledge and confidence | *“Mums have little knowledge about what breastfeeding is really like so make their choices based on what they know and have experienced –formula feeding. It’s so unusual to see breastfeeding when you are out and there are no positive role models”* (HCP)[^30^](#_ENREF_30) |
|  | Influence of peers/family | *“Many people make breastfeeding mums feel inadequate, telling them they haven’t got enough milk, which leads to confusion and lack of confidence in their ability to breastfeed.”* (PCP)[^30^](#_ENREF_30) |
|  | Time constraints | *“It’s not always just a lactation visit. There is so much to cover”* (Nurse)[^12^](#_ENREF_12) |
|  | Gap in provision of BF care services | *“We go in generally after the 10th day and quite often the mums are topping up and have been told their milk's not strong enough, (or) there isn't enough...”* (HV)[^43^](#_ENREF_43) |
|  | PCPs vary in their approach | *“I have had 5 different people tell me 5 different things and you’re going to be another one.’ I had one woman say that to me and you just have to calm down in these situations and it is not always taken well”* (Nurse)[^12^](#_ENREF_12) |
|  | Lack of BF training | *“…I got it in my course [basic training] ‘by osmosis’ picking up bits of advice...In spite of this, the course had restricted access, and there was no plan to use the skills of graduates in any structured way*” (Midwife)[^44^](#_ENREF_44) |
| Barriers to providing anticipatory  guidance:  practitioner  level | Deficit in knowledge | *“If I was more clued up on what information to give I would feel happy to do that so it would boil down to education I think”* (Nurse)[^31^](#_ENREF_31) |
|  | Lack of familiarity with guideline content | *“...I know what [the dietary] recommendations are, but only on a broad basis and not on a “well...show me your dietary diary and let’s see if ...” I’m not going to presume to be a dietician... I don’t have that training”* (Physician)[^23^](#_ENREF_23) |
|  | Lack of skills | *“That (counselling about increasing physical activity and decreasing sedentary activity] doesn’t seem as overwhelming to me as counseling about diet does.”* (Physician)[^23^](#_ENREF_23) |
|  | Uncertainty about identifying infants at risk of developing obesity | *“I have no problems at identifying obese sort of toddler, bigger than that, but really with babies I would feel quite concerned about saying that a child was obese as a baby”* (HV)[^21^](#_ENREF_21) |
|  | Disagreement with guideline content/ evidence/ usefulness | *“I’m challenged by] the lack of standardization of what defines ‘what is obesity’ in that age because as far as I know there is not a standard definition.’’* (PCP)[^1^](#_ENREF_1)  *“…we do see a lot of kids from four to five months starting to show that great interest in food and wanting to eat…So we have to sort of treat every child as an individual…So we can’t say six months 100 % for every child”* (Nurse)[^13^](#_ENREF_13) |
|  | Lack of confidence | *“There are certain times you’d want to open the door [discussing food and weight] and sometimes you don’t open the door because you don’t know what lies behind that.”* (Nurse)[^36^](#_ENREF_36)  *“…It’s something about not just informing or giving knowledge but how do you actually do it in ways that people will then integrate it?”* (Public Health Nurse)[^13^](#_ENREF_13) |
|  | Prioritise family centred care | *“I am not the kind of person who closely follows protocols. I try to tailor my advices to what fits into the parent’s lifestyle”* (Physician)[^25^](#_ENREF_25) |
|  | Belief: PCP’s prevention efforts have little impact | *“...We’re only seeing them for fifteen minutes. How much can we really get accomplished? And there’s a lot do to prevent obesity.”* (Clinician)[^10^](#_ENREF_10)  “*I don't know whether we're making any great changes or having any great influence on their decisions… Probably because the amount of time that we get to spend with people.”* (Nurse)[^2^](#_ENREF_2) |
|  | Belief: risk of harm to relationship with family | *‘‘I think sometimes the provider may defer the conversation or touch on things lightly in an attempt to maintain rapport so they can continue to have an ongoing conversation in the future.’’* (PCP) [^1^](#_ENREF_1) |
|  | Uncertainty about role in prevention of childhood obesity | *“I personally would never [address childhood obesity]. I would have them talk to their doctor about that. I would never say, ‘because he is at this weight, your child is unhealthy.”* (Nurse) [^36^](#_ENREF_36)  *“Who should deliver it, well the health visitor would either deliver it herself or she would know where parents could go to get the information”* (Physician) [^31^](#_ENREF_31) |
|  | Normalisation of mild overweight | *“It is normal to be slightly overweight, really. We have changed our values somewhat. One doesn’t react quite as quickly as before when children are chubby”* (Nurse) [^20^](#_ENREF_20) |
| Barriers to providing anticipatory  guidance:  Family/ parent level barriers  Family/ parent level barriers | Fear of  offending  parents | *I have learned to be careful with what I say to the parents. It’s difficult to know how to present concern. You don’t want to offend the parent. Already from the start, weight is a bit sensitive for the parents"* (Nurse) [^26^](#_ENREF_26)  *“Parents getting offended (often they are overweight, too) … parents start crying and get offended”* (Nurse) [^6^](#_ENREF_6) |
|  | Parental resistance/lack of concern/lack of motivation | *“However, when parents are strongly opinionated, sometimes consultation is not more than advice, because you can’t force parents to follow your advice.”* (Nurse) [^25^](#_ENREF_25)  *“If they have got their barriers up or, you know, their ears are closed, and you are bashing your head against a brick wall quite often, just – you know they don’t want to hear it. They don’t want to know about it”* (Nurse) [^35^](#_ENREF_35) |
|  | Beliefs and lifestyles of parents who are living with overweight | *“The largest risk is probably when obese parents do not consider overweight problematic. Many suppose that because of their own overweight, their children will also be overweight. These parents will not change their lifestyle.”*  (Nurse) [^25^](#_ENREF_25) |
|  | Parents’ knowledge and skills | *"…I think poor socio-economic groups tend to have less knowledge on diet, as well as less money too, they think that a healthy diet is more expensive. I do feel that a lot of young parents now haven’t learnt how to cook, from basics"* (Nurse) [^31^](#_ENREF_31) |
|  | Parents’ mis-perception of healthy infant weight | *“Usually most of them associate a fat baby as a healthy baby, and they don’t perceive the baby as gaining too much weight. Really, everyone is marvelling at that baby and saying ‘Oh, what a big baby. Look at those legs, they’re so big.’ So they see that as something really good.”* (Nurse/ nutrition expert)[^50^](#_ENREF_50) |
|  | Influence of peers/ grandparents | *“You’re not going to get them to leave their social support group…If there are four other women around saying, ‘that’s not how we do it...’ Even if you really want to do [what the provider says], you’re going to feel bad because they’re there all the time.”* (Practitioner)[^36^](#_ENREF_36) |
|  | Sociocultural norms influence perceptions | *"Well there are definitely in the Asian community it’s definitely a sign of prosperity to have nice chubby children"* (HV)[^21^](#_ENREF_21) |
|  | Parents have other complex problems to deal with | *‘But you have a few families...where the problem with the child’s overweight is just one little problem together with all the other problems in the family...In this case it’s not so easy”* (Nurse)[^19^](#_ENREF_19) |
|  | Parent/family Socioeconomic conditions | *“…the thing is that we live in a society where the cheapest foods available to us are the most calorie dense and so when you don’t have access to good healthy choices it’s tough and you have to make that extra effort”* (PCP)[^5^](#_ENREF_5) |
| Barriers to providing anticipatory  guidance:  Organisational level | Time constraints/ | *“Another question is whether there is enough consultation time. There are a lot of topics to which attention has to be paid during the consultation with parents. Time is a restrictive factor…”* (Physician)[^25^](#_ENREF_25) |
|  | Lack of role support | *"Yes, we started, but then we realized…that with this amount of resources, is it possible to do a qualitatively good job? In our opinion it’s necessary to increase the budget, making it possible for us to offer the families the good nursing they deserve…and we figured - status quo, we cannot do it."* (Nurse)[^8^](#_ENREF_8) |
|  | Lack of training | *“Like I mentioned I never had any training, but I’m giving advice, and I’m sure there’s lots of other people in the same position.”* (Physician)[^31^](#_ENREF_31) |
|  | Lack of resources | *‘‘If there were a tool more readily available for beyond just looking at percentages and the rate of growth on growth charts. If there were maybe not BMI but maybe rate of weight gain so you could say it would help keep on the radar.’’* (PCP)[^1^](#_ENREF_1) |
|  | Lack of united coherent approach | *“Actually, there is no cooperation with paediatricians and family doctors concerning the topic of*  *overweight*” (Physician)^[25](#_ENREF_25)^ |
|  | Lack of support from other PCP groups | *“It is really difficult and unfortunate for us, because it would matter so much if the doctor did it. The doctor has a great power. And if the doctor said ‘It’s important that you’ll get some lifestyle conversations with the health visitor’, it would be so much easier for us to get the message through to the family”* (HV)[^9^](#_ENREF_9) |
|  | Lack of opportunity for contact | *“… traditionally we see kids up to the two-year old because that’s their last inoculation or … eighteen months. So we don’t actually see them…. until they go to school … there’s that gap in their care...”* (Physician)[^10^](#_ENREF_10) |
|  | Lack of continuity of care | *“If you had someone whom you could see a couple of times in a row, then you could build up a rapport…"* (Nurse)[^12^](#_ENREF_12) |
|  | Limited access to community programs/ | *“Yes, the dietician or physiotherapist...However, I don’t know which programs they have for example to stimulate physical activity for the young child.”* (Physician)[^25^](#_ENREF_25) |

**Table S4. Overview of the facilitators with indicative quotes.**

[Abbreviations: BMI= body mass index; PCP= primary care practitioner]

| Category | Finding | Indicative quotes |
| --- | --- | --- |
| Facilitators:  practitioner level | Knowledge and confidence | *“We didn’t know if this was a good way to do it. We didn’t know if this was the final way. We wanted to make sure there was room to improve, and we wanted everyone to focus on the quality assurance this would lead to.’’* (Nurse)[^8^](#_ENREF_8) |
|  | Communication skills | *“Yes, first of all you want to do it in a respectful manner, because many of the parents feel they have failed when they see the percentile pointing in the wrong direction…But we've been thinking and reflecting a lot on which methods to use to motivate the parents, and also to explain…. I think these guidelines are so useful in that way….”* (Nurse)[^8^](#_ENREF_8) |
|  | Ability to use tools to aid communication | *“But I feel that when you have the BMI chart, you have so much to benefit there, that you can show it, and we can truly say that now it has increased. No, you can look at it and talk about it, and talk a little about what you could change”* (Nurse)[^20^](#_ENREF_20) |
|  | Positive beliefs about role | *“I’d say we have a lot of contact with families certainly in the first six months around feeding and moving onto weaning and then early young children’s diet forms quite a significant part of my role.”* (Health Visitor)[^21^](#_ENREF_21) |
|  | Belief: positive feelings about role | *“I do not think that I have avoided it, because my task it is to ensure that the children feel as well as possible and get a good start in life…You know, a lot happens between the ages of 2& 1/2 years and 6 years, and if I see something, then it’s my responsibility.”* (Nurse)[^20^](#_ENREF_20) |
|  | Positive relationship with family | *“And you have to work also on your relationship, how good relationship you have with the family depending upon the degree of how much you can come down on them about it...”* (Nurse)[^35^](#_ENREF_35) |
| Facilitator: Parent level | Receptive parents | *“They embrace what you talk about, changing the diet and trying to assimilate the tips and advice that I have given…The easiest ones are the parents who say ‘help me’. They’re definitely the easiest.”* (Nurse)[^20^](#_ENREF_20) |
| Facilitator:  Organisation  level  Facilitator:  Organisation  level | Perception of role support | *“The DGP [Division of General Practice] came through and gave us support to set it HKC [Healthy Kids Check] up. We have a template from them...and also training at the Division so I’m fairly confident in what I’m doing”* (Nurse)[^18^](#_ENREF_18) |
|  | Provision of training | *“Training has helped confidence”* (Nurse)[^4^](#_ENREF_4) |
|  | United approach | *“And what we’re working hard on, everyone who works at the CHC, and those who work in the children’s team, is that we try to talk the same language, that we do not say different things, because it gives a sense of insecurity*” (Nurse)[^20^](#_ENREF_20) |
|  | Adequate resources | *“If we had somebody who was able to sit down and spend a focused amount of time with the parent and the child in the [clinic] setting, it seems like it would be more effective than me trying to do everything at the time of the well child exam"* (PCP)[^23^](#_ENREF_23) |
|  | Resource and training needs | *“I think assessing children’s diets and the children’s BMI...would actually be quite good (to have) a bit more background... healthy eating recommendations for children... physical activity recommendations . . . and then perhaps some strategies to encourage families to adopt healthy lifestyles as well”* (Nurse)[^18^](#_ENREF_18) |
|  | Professional autonomy | *“I think that we’d be more effective if we could just find out where the person is that day and not be required in the 10 minutes we have with them to go through all the information… and we could be more effective in our timing if we counselled more on where the person was, instead of what we were required to cover ”* (Healthcare provider)[^50^](#_ENREF_50) |

References (listing the 50 studies included in the review).

1.Andersen LM, Thompson DA. Managing Excessive Weight Gain in Children< 2 Years of Age: Interviews with Primary Care Providers. *Childhood Obesity*. 2020;16(5):332-9.

2.Cheng H, Eames-Brown R, Tutt A, et al. Promoting healthy weight for all young children: a mixed methods study of child and family health nurses’ perceptions of barriers and how to overcome them. *BMC Nursing*. 2020;19(1):1-14.

3.Belay B, Frintner MP, Liebhart JL, et al. US Pediatrician Practices and Attitudes Concerning Childhood Obesity: 2006 and 2017. *Journal of Pediatrics*. 2019;211:78-84.e2.

4.Moir C, Jones V. Experience of nurses measuring preschool body mass index for the Health target: Raising Healthy Kids. *Journal of Primary Health Care*. 2019;11(3):275-82.

5.Kracht CL, Sisson SB, Kerr K, et al. Health Care Provider’s Role in Obesity Prevention and Healthy Development of Young American Indian Children. *Journal of Transcultural Nursing*. 2019;30(3):231-41.

6.Tanda R, Beverly EA, Hughes K. Factors associated with Ohio nurse practitioners’ childhood obesity preventive practice patterns. *Journal of the American Association of Nurse Practitioners*. 2017;29(12):763-72.

7.Dera-de Bie EG, Brink-Melis W, Jansen M, Gerver WJ. Characteristics of child health care practitioners in overweight prevention of children. *Applied Nursing Research*. 2016;29:157-62.

8.Nordstrand A, Fridlund B, Sollesnes R. Implementation of national guidelines for the prevention and treatment of overweight and obesity in children and adolescents: a phenomenographic analysis of public health nurses' perceptions. *Int J Qual Stud Health Well-being*. 2016;11(1):31934.

9.Ditlevsen K, Reventlow S, Nielsen A. From policy to reality: early overweight, structural barriers, and the allocation of responsibility in the Danish health care system. *Critical Public Health*. 2016;26(5):566-77.

10.Bourgeois N, Brauer P, Simpson JR, Kim S, Haines J. Interventions for prevention of childhood obesity in primary care: a qualitative study. *Canadian Medical Association Journal*. 2016;4(2):E194-9.

11.Nelson JM, Vos MB, Walsh SM, O'Brien LA, Welsh JA. Weight management-related assessment and counseling by primary care providers in an area of high childhood obesity prevalence: current practices and areas of opportunity. *Childhood Obesity*. 2015;11(2):194-201.

12.McLelland G, Hall H, Gilmour C, Cant R. Support needs of breast-feeding women: Views of Australian midwives and health nurses. *Midwifery*. 2015;31(1):e1-e6.

13.Laws R, Campbell KJ, Pligt P, et al. Obesity prevention in early life: an opportunity to better support the role of Maternal and Child Health Nurses in Australia. *BMC Nursing*. 2015;14(1):1-14.

14.Chelvakumar G, Levin L, Polfuss M, Hovis S, Donohoue P, Kotowski A. Perception and documentation of weight management practices in pediatric primary care. *Wisconsin Medical Journal*. 2014;113(4):149-53.

15.Pound CM, Williams K, Grenon R, Aglipay M, Plint AC. Breastfeeding knowledge, confidence, beliefs, and attitudes of Canadian physicians. *Journal of Human Lactation*. 2014;30(3):298-309.

16.Bonnet J, George A, Evans P, Silberberg M, Dolinsky D. Rethinking obesity counseling: having the French Fry Discussion. Journal of Obesity [Internet]. 2014. Available from: https://www.hindawi.com/journals/jobe/2014/525021/.

17.Lowenstein LM, Perrin EM, Campbell MK, Tate DF, Cai J, Ammerman AS. Primary care providers' self-efficacy and outcome expectations for childhood obesity counseling. *Childhood Obesity*. 2013;9(3):208-15.

18.Robinson A, Denney‐Wilson E, Laws R, Harris M. Child obesity prevention in primary health care: investigating practice nurse roles, attitudes and current practices. *Journal of Paediatrics and Child Health*. 2013;49(4):E294-E9.

19.Ljungkrona-Falk L, Brekke H, Nyholm M. Swedish nurses encounter barriers when promoting healthy habits in children. *Health Promotion International*. 2014;29(4):730-8.

20.Regber S, Mårild S, Hanse JJ. Barriers to and facilitators of nurse-parent interaction intended to promote healthy weight gain and prevent childhood obesity at Swedish child health centers. *BMC Nursing*. 2013;12(1):1-11.

21.Redsell SA, Swift JA, Nathan D, Siriwardena AN, Atkinson P, Glazebrook C. UK health visitors' role in identifying and intervening with infants at risk of developing obesity. *Maternal & Child Nutrition*. 2013;9(3):396-408.

22.Isma GE, Bramhagen A-C, Ahlstrom G, Östman M, Dykes A-K. Obstacles to the prevention of overweight and obesity in the context of child health care in Sweden. *BMC Family Practice*. 2013;14(1):1-10.

23.Findholt NE, Davis MM, Michael YL. Perceived barriers, resources, and training needs of rural primary care providers relevant to the management of childhood obesity. *The Journal of Rural Health*. 2013;29(s1):s17-s24.

24.Bohman B, Eriksson M, Lind M, Ghaderi A, Forsberg L, Rasmussen F. Infrequent attention to dietary and physical activity behaviours in conversations in Swedish child health services. *Acta Paediatrica*. 2013;102(5):520-4.

25.Dera de Bie E, Jansen M, Gerver WJ. Inhibiting Factors in the prevention of overweight in infants: An explorative qualitative study among child healthcare practitioners in the Netherlands. *Child Care in Practice*. 2012;18(3):193-206.

26.Isma GE, Bramhagen A-C, Ahlstrom G, Östman M, Dykes A-K. Swedish Child Health Care nurses conceptions of overweight in children: a qualitative study. *BMC Family Practice*. 2012;13(1):1-11.

27.Rausch JC, Rothbaum Perito E, Hametz P. Obesity prevention, screening, and treatment: practices of pediatric providers since the 2007 expert committee recommendations. *Clinical Pediatrics*. 2011;50(5):434-41.

28.Wethington HR, Sherry B, Polhamus B. Physician practices related to use of BMI-for-age and counseling for childhood obesity prevention: a cross-sectional study. *BMC Family Practice*. 2011;12(1):1-9.

29.Huang TT, Borowski LA, Liu B, et al. Pediatricians' and family physicians' weight-related care of children in the U.S. *American Journal of Preventive Medicine*. 2011;41(1):24-32.

30.Brown A, Raynor P, Lee M. Healthcare professionals’ and mothers’ perceptions of factors that influence decisions to breastfeed or formula feed infants: a comparative study. *Journal of Advanced Nursing*. 2011;67(9):1993-2003.

31.Redsell SA, Atkinson PJ, Nathan D, Siriwardena AN, Swift JA, Glazebrook C. Preventing childhood obesity during infancy in UK primary care: a mixed-methods study of HCPs' knowledge, beliefs and practice. *BMC Family Practice*. 2011;12(1):1-9.

32.Spivack JG, Swietlik M, Alessandrini E, Faith MS. Primary care providers' knowledge, practices, and perceived barriers to the treatment and prevention of childhood obesity. *Obesity*. 2010;18(7):1341-7.

33.Sesselberg TS, Klein JD, O'connor KG, Johnson MS. Screening and counseling for childhood obesity: results from a national survey. *The Journal of the American Board of Family Medicine*. 2010;23(3):334-42.

34.Klein JD, Sesselberg TS, Johnson MS, O'Connor KG, Cook S, Coon M. Adoption of body mass index guidelines for screening and counseling in pediatric practice. *Pediatrics*. 2010;125(2):265- 72.

35.Edvardsson K, Edvardsson D, Hörnsten Å. Raising issues about children’s overweight–maternal and child health nurses’ experiences. *Journal of Advanced Nursing*. 2009;65(12):2542-51.

36.Johnson SL, Clark L, Goree K, O'Connor M, Zimmer LM. Healthcare providers' perceptions of the factors contributing to infant obesity in a low-income Mexican American community. *Journal for Specialists in Pediatric Nursing*. 2008;13(3):180-90.

37.Woolford SJ, Clark SJ, Strecher VJ, Gebremariam A, Davis MM. Physicians' perspectives on increasing the use of BMI charts for young children. *Clinical Pediatrics*. 2008;47(6):573-7.

38.Wallace LM, Kosmala-Anderson J. Training needs survey of midwives, health visitors and voluntary-sector breastfeeding support staff in England. *Maternal and Child Nutrition*. 2007;3(1):25-39.

39.Wallace LM, Kosmala‐Anderson J. A training needs survey of doctors’ breastfeeding support skills in England. *Maternal & Child Nutrition*. 2006;2(4):217-31.

40.Serrano E, Gresock E, Suttle D, Keller A, McGarvey E. Fit WIC: attitudes, perceptions and practices of WIC staff toward addressing childhood overweight. *Journal of Nutrition Education & Behavior*. 2006;38(3):151-6.

41.Tappin D, Britten J, Broadfoot M, McInnes R. The effect of health visitors on breastfeeding in Glasgow. *International Breastfeeding Journal*. 2006;1(1):1-9.

42.Larsen L, Mandleco B, Williams M, Tiedeman M. Childhood obesity: Prevention practices of nurse practitioners. *Journal of the American Association of Nurse Practitioners*. 2006;18(2):70-9.

43.Tennant R, Wallace LM, Law S. Barriers to breastfeeding: a qualitative study of the views of health professionals and lay counsellors. *Community Practitioner*. 2006;79(5):152-6.

44.Smale M, Renfrew MJ, Marshall JL, Spiby H. Turning policy into practice: more difficult than it seems. The case of breastfeeding education. *Maternal & Child Nutrition*. 2006;2(2):103-13.

45.Perrin EM, Flower KB, Ammerman AS. Body mass index charts: useful yet underused. *The Journal of Pediatrics*. 2004;144(4):455-60.

46.Rattay KT, Fulton JE, Galuska DA. Weight counseling patterns of US pediatricians. *Obesity*. 2004;12(1):161-9.

47.Hellings P, Howe C. Breastfeeding knowledge and practice of pediatric nurse practitioners. *Journal of Pediatric Health Care*. 2004;18(1):8-14.

48.Gentile DA, Oberg C, Sherwood NE, Story M, Walsh DA, Hogan M. Well-child visits in the video age: pediatricians and the American Academy of Pediatrics' guidelines for children's media use. *Pediatrics*. 2004;114(5):1235-41.

49.Gilbert MJ, Fleming MF. Pediatricians' approach to obesity prevention counseling with their patients. *Wisconsin Medical Journal*. 2006;105(5):26-31.

50.Chamberlin LA, Sherman SN, Jain A, Powers SW, Whitaker RC. The challenge of preventing and treating obesity in low-income, preschool children: perceptions of WIC health care professionals. *Archives of Pediatrics & Adolescent Medicine*. 2002;156(7):662-8.
